# Epithelial Memory After Respiratory Viral Infection in Mice Results in Prolonged Enhancement of Antigen Presentation

**DOI:** 10.1111/all.16683

**Published:** 2025-08-07

**Authors:** Piotr P. Janas, Wouter T'Jonck, Matthew O. Burgess, Maximilian Reck, Caroline Chauché, Matthieu Vermeren, Christopher D. Lucas, Calum Bain, Robert Illingworth, Edward W. Roberts, Henry J. McSorley, Jürgen Schwarze

**Affiliations:** ^1^ Centre for Inflammation Research Institute for Regeneration and Repair, the University of Edinburgh Edinburgh Scotland; ^2^ Centre for Cardiovascular Science The University of Edinburgh Edinburgh Scotland; ^3^ CRUK Scotland Institute Glasgow Scotland; ^4^ Division of Cell Signalling and Immunology School of Life Sciences, University of Dundee Dundee Scotland

**Keywords:** epigenetic imprinting, epithelial memory, lung epithelial cells, respiratory viral infection, RSV

## Abstract

**Background:**

Viral lower respiratory tract infections (LRTIs) can reduce the severity of subsequent LRTIs but have also been linked to respiratory allergy development and exacerbation. Here, we show that viral LRTI can imprint lung epithelial cells (LECs), leading to prolonged phenotypic and functional changes.

**Methods:**

Mice were infected via intranasal administration of respiratory syncytial virus (RSV). After 28 days, LECs were isolated using cold dispase digestion followed by magnetic‐activated cell sorting. Epigenetic changes were assessed using Cleavage Under Targets and Release Using Nuclease (CUT&RUN), while transcriptional changes were evaluated using NanoString and qPCR. Flow cytometry was employed to measure cell surface major histocompatibility complex (MHC) levels, antigen uptake and processing rates, and OT‐I cell proliferation after antigen presentation.

**Results:**

We identified epigenetic and transcriptomic changes in murine LECs 28 days after RSV infection, especially impacting genes associated with MHC. Lasting upregulation of MHC‐I and MHC‐II was further increased following in vivo LPS stimulation. Importantly, MHC upregulation was associated with increased antigen uptake and processing, as well as increased antigen presentation to T cells.

**Conclusions:**

Our data demonstrate that RSV can induce prolonged upregulation of antigen presentation by LECs, with the potential to facilitate local T cell responses to microbial antigens and allergens and to enhance immunity or in susceptible hosts respiratory allergy.

## Graphical abstract

- RSV infection induces lasting epigenetic and transcriptional changes in LECs, upregulating MHC‐I and MHC‐II, further amplified by LPS exposure.
- This is accompanied by increased antigen uptake, processing, and presentation.
- These persistent alterations may influence immune responses, potentially impacting both immunity to infection and respiratory allergy development.

Abbreviations: IF, immunofluorescent; LECs, lung epithelial cells; LPS, lipopolysaccharide; MHC, major histocompatibility complex; PBS, phosphate buffered saline; RSV, respiratory syncytial virus.

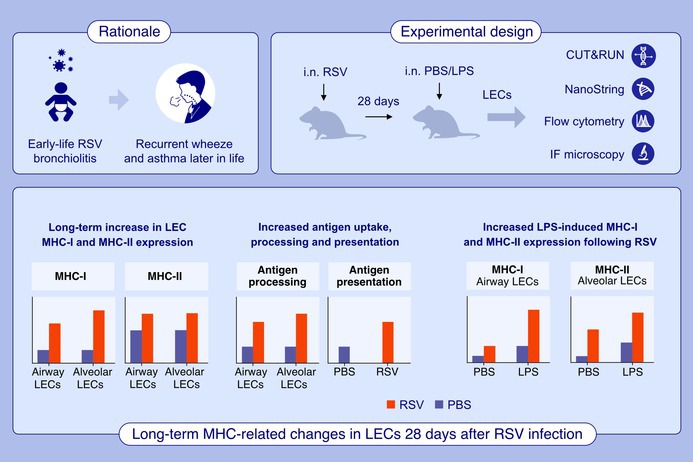

## 1 Introduction

Viral lower respiratory tract infections (LRTI) including by rhinovirus [1], human metapneumovirus [2] and respiratory syncytial virus (RSV) [3, 4] are linked to the development and exacerbation [5, 6] of allergic airway disease but can also protect from subsequent respiratory infections [7, 8]. While multiple genetic, epigenetic, and environmental factors predisposing to allergic airway disease development have been described [9, 10], the underlying cellular and molecular mechanisms are still unclear. Historically, respiratory epithelial cells were viewed primarily as a relatively inert barrier at the interface of environment and host; however, in recent years, their importance in orchestrating initial innate immune responses has been recognized [11, 12]. Given their relatively long lifespan [13] and findings such as those by Byers et al. (2013), which demonstrated that lung epithelial progenitor cells maintain IL‐33 production after Sendai virus infection [14], and Naik et al. (2017), which showed skin epithelial cells retain a memory of inflammation [15], we hypothesized that viral LRTI can induce prolonged changes in lung epithelial cells (LECs). These changes might affect responses to future infections and allergen exposure. We used an established mouse model of RSV infection [16, 17, 18] to investigate prolonged changes in LECs following viral clearance. Here, we reveal epigenetic and transcriptomic changes in genes primarily associated with major histocompatibility complexes (MHC) and antigen processing that persist for a prolonged period after RSV infection. LECs also maintained elevated levels of both MHC‐I and MHC‐II, with further enhancement after stimulation with lipopolysaccharide (LPS). These prolonged changes were associated with increases in antigen uptake, processing, and presentation to T cells.

## 2 Materials and Methods

### 2.1 RSV Stock and Immunoplaque Assay

RSV A2 (kindly provided by Dr. James Harker, Imperial College London) was expanded in Hep‐2 cells as previously described [19]. Once a 10%–20% drop in confluency and syncytia were observed throughout, cells were sonicated, and the virus was harvested. RSV titers were assessed using an immunoplaque assay. Plaque‐forming units (PFU/ml) were calculated based on the average plaque count and a dilution factor.

### 2.2 Animals and Animal Procedures

Wild‐type female BALB/c and C57BL/6J mice were procured from Charles River Laboratories. Mice were acclimatized for 1 week before experimental use. OT‐I mice were imported from the CRUK Beatson Institute, Glasgow. Both male and female OT‐I mice ranging from two to 6 months old were used for OT‐I cell harvest. Mice were housed in individually ventilated cages. Animal work was carried out under the regulations of the Animals (Scientific Procedures) Act 1986. All procedures were approved by the University of Edinburgh Animal Welfare and Ethical Review Board and performed under UK Home Office licenses with institutional oversight performed by qualified veterinarians. UK Home Office project license to JS, number PP4544912. ARRIVE 2.0 guidelines were followed where applicable.

6‐week‐old BALB/c or C57BL/6J mice were administered 50 μL of 5 × 10^6^/ml RSV A2 intranasally under light anesthesia (inhaled isoflurane). Alternatively, mice were administered 50 μL of PBS (w/o Ca^2+^ and Mg^2+^) or UV‐irradiated RSV (using SPECTROLINKER XL‐1500 at 2 J/cm^2^). After 28 days, mice were either processed as described below or intranasally administered 50 μL PBS (w/o Ca^2+^ and Mg^2+^) or 10 μg of LPS (Sigma‐Aldrich, USA, *E. coli* O111:b4) in 50 μL PBS either 6 h or 24 h before further processing as described below.

### 2.3 Murine Lung Harvest and Processing

Murine lungs were harvested and processed as previously described [20]. In brief, lungs were inflated with ice‐cold 1.5‐2 mL enzyme mix (DMEM/F12 (Gibco, USA) + 100 U/mL Pen/Strep (Gibco, USA) + 2 mg/mL Dispase II (Sigma‐Aldrich, USA) + 0.1 mg/mL DNaseI (Sigma‐Aldrich, USA)). Lungs were then incubated at 4°C–6°C for 20 h. After incubation, lungs were passed through 70 and 30 μm strainers, and red blood cells (RBC) were lysed for 2 min using ACK RBC lysis buffer (Gibco, USA). Cells were resuspended in MACS buffer (PBS w/o Mg^2+^ and Ca^2+^ +0.5% bovine serum albumin (BSA) (Sigma‐Aldrich, USA) + 2 mM EDTA (Gibco, USA) + 100 U/mL Pen/Strep (Gibco, USA)). Cells were then either resuspended in the appropriate media for in vitro culture (discussed below) or incubated with 5 μL/1 mL/lung antimouse CD16/32 antibody (BioLegend, USA) for 30 min at 4°C before further processing.

### 2.4 Isolation of Lung Epithelial Cells

LECs were MACS‐isolated as previously described [20]. In brief, up to 10^7^ cells were incubated with 5 μL anti‐CD31 microbeads and 10 μL of anti‐CD45 microbeads (Miltenyi Biotec, Germany), followed by LS column magnetic isolation. Flowthrough cells were incubated with 15 μL of anti‐EpCAM microbeads (Miltenyi Biotec, Germany) followed by magnetic isolation with MS columns. CD45‐CD31‐EpCAM+ LECs were then flushed out from MS columns with a plunger, and their purity (> 97%) was assessed using flow cytometry (Figure S1).

### 2.5 Cleavage Under Targets and Release Using Nuclease (CUT&RUN)

2 × 10^5^ of MACS‐sorted murine LECs from a pool of three mice were used per single CUT&RUN reaction. Unless specified otherwise, samples were kept at 4°C. Cells were washed twice using PBS with 600 × g centrifugation steps in between each wash. Cells were then incubated in 10% Triton X‐100 (Sigma‐Aldrich, USA) for 10 min to isolate nuclei. Nuclei were pelleted following 1300 × g centrifugation for 5 min and resuspended in wash buffer. Following nuclear isolation, a CUT&RUN protocol [21] was followed. In brief, nuclei were bound to BioMag Plus Concavalin A beads (Bangs Laboratories, USA) and resuspended in antibody buffer containing either anti‐H3K27ac (1:100, Active Motif, USA), anti‐H3K4me3 (1:100, Sigma‐Aldrich, USA) or normal rabbit IgG (1:100, Cell Signalling Technology, USA). Following overnight incubation, nuclei were washed thrice with wash buffer and incubated with protein A/G‐MNase (VIB Protein Core, kindly provided by Prof. Martin Guilliams) for 1 h while rotating. Chromatin digestion and release were performed using the high Ca^2+^/low salt method followed by phenol/chloroform extraction. DNA was stored at −20°C until further processing. KAPA Prep Kit (Roche, Switzerland) and KAPA UDI adapter kit (Roche, Switzerland) were used for library preparations per manufacturer's instructions. In brief, end repair 5′ phosphorylation (20°C, 30 min), dA‐tailing (58°C, 45 min) and adapter ligation (20°C, 60 min) were followed by un‐ligated adapter removal with 1.1 × volume of AMPure XP beads (Beckman Coulter, USA). Libraries were amplified over 12 cycles in the thermocycler using KAPA HiFi HotStart ReadyMix and 5 μM Pre‐LM‐PCR Oligo 1 & 2 (Roche, Switzerland). Following amplification, another 3‐step adapter removal with AMPure XP beads was carried out. Samples were quality controlled using LabChip GX DNA High Sensitivity (Caliper Life Sciences, USA) according to manufacturer's instructions.

Libraries were then submitted to Azenta Life Sciences for next generation sequencing using 2 × 150bp configuration at 5 × 10^6^ reads per sample. FASTQ sequencing files were uploaded to Galaxy platform [22] for QC using FastQC [23] and sequence trimming using Trimmomatic [24], followed by sequence alignment to mm10 mouse reference genome using Bowtie2 [25]. Next, the bamCoverage [26] tool was used to generate bin size 5 read coverage .bam files, followed by peak calling using Sparse Enrichment Analysis for CUT&RUN (SEACR) [27]. Default settings were used for all Galaxy platform tools. Bowtie2 and SEACR files were exported and analyzed further using R Studio R4.3.1. Defined package [28] (release. 3.14) to identify differentially bound sites (DBS). When using dba.count function duplicate reads were not removed (bRemoveDuplicates = F), peaks were resized to center around their summits (summits = T) and no peaks were filtered out based on read count (filter = 0). Normalization was performed using the dba.normalize command (method = DBA_DESEQ2, normalize = DBA_NORM_NATIVE). Contrast between the groups was performed using dba.analyze with problematic regions removed using ENCODE Blacklist database [29] (bBlacklist = T), while statistical discovery of significant DBS was carried out using DESeq2 [30] (method = DBA_DESEQ2). All other parameters in the DiffBind package were set to default. Gene annotation and genomic annotation (TxDb.Mmusculus.UCSC.mm10.knownGene) were carried out using ChIPSeeker [31] (release 1.4), while gene ontology (GO) analysis was carried out using ClusterProfiler [32] (release 4.12). DBS within +/−3 kb of TSS were visualized using deeptools (v.3.5.4). bigWig files were generated from bam files using bamCoverage (−binSize 10 ‐normalizeUsing RPGC ‐effectiveGenomeSize 2,150,570,000 –extendReads). Scores of genomic regions in proximity to differentially accessible peaks were calculated using computeMatrix (−referencePoint TSS, ‒afterRegionStartLength 3000 ‒beforeRegionStartLength 3000) and heatmaps were visualized using plotHeatmap.

### 2.6 RNA Extraction

RNA was extracted as previously described [20]. In brief, 100 μL of bromochloropropane (Sigma‐Aldrich, USA) was added to cells treated with TRizol (Life Technologies, USA), followed by 16,000 × g at 4°C for 20 min centrifugation and RNA precipitation using isopropanol. RNA pellets were then washed three times using 70% EtOH. After determining RNA concentration, samples were normalized using RNase‐free H_2_O, followed by treatment with DNase I (QIAGEN, Germany) as per the manufacturer's instructions to remove genomic DNA.

### 2.7 NanoString

All RNA samples were determined to be of high quality by RNA 6000 Pico Assay (Agilent, USA), with > 80% DV200. RNA from two mice was pooled to generate a single sample. Each sample was normalized to 20 ng/μl, and 8 μL/sample of RNA was profiled using Nanostring nCounter Mouse Immunology Panel according to the manufacturer's instructions. NanoString data were analyzed using “NanoTube” package [33] (release 3.18) in RStudio R4.3.1 as previously described [34]. The bgPval was set to 0.01 and the number of unwanted factors (k) was set to 3, while the remaining normalization parameters were set to default. Results are considered significant for FDR values of < 0.05.

### 2.8 qPCR

qPCRs using TaqMan probes (Table 1) were performed as previously described [20]. Each sample was normalized to the appropriate endogenous control within the reaction, and log2FC was calculated using the ΔΔCt method. Appropriate endogenous controls were selected using the “NormFinder” package [35] (release 0.1.2). *Oaz1* and *Gapdh* were identified as the most stably expressed endogenous controls between experimental groups (Table S1). *Oaz1* was used for low‐expressed targets, while *Gapdh* was used for highly expressed targets. Custom primers were used for measuring RSV L‐gene. Forward' RSV L‐gene primer: GAACTCAGTGTAGGTAGAATGTTTGCA. Reverse 3′ L‐gene primer: TTTCAGCTATCATTTTCTCTGCCAAT. RSV L‐gene TaqMan probe: (FAM) TTTGAACCTGTCTGAACATTCCCGGTT (TAMRA). qPCR data are presented as log2FC relative to the PBS sample.

**TABLE 1:** TaqMan qPCR probe details.

Target	Species	Probe	Assay ID	Catalogue number	Endogenous control
*Gapdh*	Mouse	VIC‐MGB	Mm99999915_g1	4,448,489	NA
*Oaz1*	Mouse	VIC‐MGB	Mm07307469_g1	4,448,489	NA
*H2‐DMb2*	Mouse	FAM‐MGB	Mm00783707_s1	4,331,182	*Oaz1*
*B2m*	Mouse	FAM‐MGB	Mm00437762_m1	4,331,182	*Gapdh*
*H2‐Ab1*	Mouse	FAM‐MGB	Mm00439216_m1	4,331,182	*Gapdh*
*H2‐Eb1*	Mouse	FAM‐MGB	Mm00439221_m1	4,331,182	*Oaz1*
*Casp4*	Mouse	FAM‐MGB	Mm00432304_m1	4,331,182	*Oaz1*
*Psmb9*	Mouse	FAM‐MGB	Mm00479004_m1	4,331,182	*Oaz1*
*Tap1*	Mouse	FAM‐MGB	Mm00443188_m1	4,331,182	*Oaz1*

### 2.9 Confocal Immunofluorescent Microscopy

Murine lungs were inflated using 0.8 mL of a 1:1 PBS/OCT mixture and placed in antigenfix (Diapath, Italy) for 45 min at RT. Lungs were then washed twice using PBS and placed in a 34% sucrose solution for 24 h at 4°C. Lungs were snap frozen in OCT and sliced (7 μm sections). Following rehydration, sections were incubated in 100 μL of blocking buffer (Tween‐20, 2% BSA, 5% FBS (LabTech, UK), 2% mouse serum (Thermo Fisher Scientific, USA) and 5% normal goat serum (Thermo Fisher Scientific, USA) in PBS) for 1.5 h at RT in a humidity chamber. Fluorochrome‐conjugated antibodies (1:400 anti‐EpCAM‐AF594, 1:200 anti‐MHC‐I‐AF647 and 1:400 anti‐MHC‐II‐AF488 (BioLegend, USA)) were diluted in blocking buffer, and 100 μL of staining mix was placed on each section for 1 h at RT. Slides were imaged using a Leica SP8 confocal microscope with a 20 × /0.75 objective without immersion or a 40 × /1.3 objective with oil immersion. A minimum of 3 fields of view were captured per section. Images were imported into Fiji, and nuclear StarDist [36] segmentation refined by EpCAM expression stratifying to the airway (EpCAM^high^) and alveolar (EpCAM^low^) regions was carried out (Figure S2). The mean fluorescence intensity of MHC‐I (AF647) and MHC‐II (AF488) channels was measured for each section. Segmentation was visually assessed, and each section with incorrect segmentation was eliminated from further analysis (in total 7 sections were removed out of the 84 sections imaged).

### 2.10 Flow Cytometry

Staining for flow cytometry analysis was performed as previously described [20]. In brief, following incubation with anti‐CD16/32 antibody, cells were stained using a LIVE/DEAD fixable near‐IR stain (Invitrogen, USA), followed by staining with the appropriate antibody cocktail (Table 2). For intracellular TLR4 staining, the eBioscience Foxp3/Transcription Factor Staining Buffer Set (Invitrogen) was used according to the manufacturer's protocol. Samples were unmixed (with multiple autofluorescence extraction) using Cytek Aurora with Cytek SpectroFlo 3.3 and analyzed using De Novo Software FCSexpress 7. The gating strategy involved debris exclusion (side scatter/forward scatter—SSC‐H/FSC‐H), followed by singlet selection (FSC‐H/FSC‐A), RBC exclusion (SSC‐H/SSC‐B‐H) and dead cell exclusion (LIVE/DEAD Fixable Near‐IR/FSC‐H), followed by further experiment‐specific gating (Figure S3, Figure S4). Flow cytometry data are presented as fold change relative to the average median fluorescence intensity (MFI) of PBS samples. We validated our EpCAM stratification strategy to airway and alveolar LECs using CD49f (airway epithelial marker [37]), MHC‐II (pneumocyte marker [38]), and CD24 (conducting airways marker [39]) (Figure S5).

**TABLE 2:** Flow cytometry antibodies.

Antibody name	Supplier	Host species	Antibody type	Clone	Catalogue number	Antibody Registry ID	Dilution
Anti‐CD45 Pacific Blue	BioLegend	Rat	Monoclonal	S18009F	157,212	AB_2876534	1:200
Anti‐CD45 AF700	BioLegend	Rat	Monoclonal	S18009F	157,210	AB_2860730	1:200
Anti‐CD31 BV605	BioLegend	Rat	Monoclonal	390	102,427	AB_2563982	1:600
Anti‐CD31 BV421	BioLegend	Rat	Monoclonal	390	102,423	AB_2562186	1:300
Anti‐EpCAM PE/Dazzle594	BioLegend	Rat	Monoclonal	G8.8	118,236	AB_2632777	1:300/1:150
Anti‐EpCAM FITC	BioLegend	Rat	Monoclonal	G8.8	118,207	AB_1134106	1:200
Anti‐EpCAM BV605	BioLegend	Rat	Monoclonal	G8.8	118,227	AB_2563984	1:300
Anti‐MHC‐I (H2kd/H2Dd) AF647	BioLegend	Mouse	Monoclonal	34–1‐2S	114,712	AB_493063	1:500
Anti‐MHC‐I (H2Kb) AF647	BioLegend	Mouse	Monoclonal	AF6‐88.5	116,511	AB_492918	1:500
Anti‐ I‐A/I‐E (MHC‐II) AF488	BioLegend	Rat	Monoclonal	M5/114.15.2	107,616	AB_493523	1:500
Anti‐MHC‐II PerCP‐Cy5.5	BioLegend	Rat	Monoclonal	M5/114.15.2	107,625	AB_2191071	1:500
Anti‐TLR4 PE‐Cy7	BioLegend	Rat	Monoclonal	MTS510	117,609	AB_2044019	1:125
Anti‐CD3 BV750	BioLegend	Rat	Monoclonal	17A2	100,249	AB_2734148	1:100
Anti‐CD4 BV605	BioLegend	Rat	Monoclonal	RM4‐5	100,547	AB_11125962	1:200
Anti‐CD8a PerCP/Cy.5.5	BioLegend	Rat	Monoclonal	53–6.7	100,733	AB_2075239	1:100
Anti‐CD69 PE	BioLegend	Hamster	Monoclonal	H1.2F3	104,507	AB_313110	1:100
Anti‐CD24 PE/Cyanine7	BioLegend	Rat	Monoclonal	M1/69	101,821	AB_756048	1:200
Anti‐CD49f PE‐Dazzle594	BioLegend	Rat	Monoclonal	GoH3	313,625	AB_2616782	1:100
CD19 PB	BioLegend	Rat	Monoclonal	6D5	115,526	AB_493341	1:50
CD26 PE‐Cy7	BioLegend	Rat	Monoclonal	H194‐112	137,809	AB_2564312	1:125
FceR1 AF647	BioLegend	Hamster	Monoclonal	MAR1	134,309	AB_1626097	1:50
CD3 BV750	BioLegend	Rat	Monoclonal	17A2	100,249	AB_2734148	1:250
CD64 BV711	BioLegend	Mouse	Monoclonal	X54‐5/7.1	139,311	AB_2563846	1:250
CD11b BV570	BioLegend	Rat	Monoclonal	M1/70	101,233	AB_1089694	1:125
CD8a PerCP‐Cy5.5	BioLegend	Rat	Monoclonal	53–6.7	100,733	AB_2075239	1:125
SiglecF PE/Dazzle594	BioLegend	Rat	Monoclonal	S17007L	155,529	AB_2890716	1:125
CD172a (sirpa) FITC	BioLegend	Rat	Monoclonal	P84	144,005	AB_11204432	1:50
MerTK PE	BioLegend	Rat	Monoclonal	2B10C42	151,505	AB_2617037	1:125
XCR1 APC	BioLegend	Mouse	Monoclonal	ZET	148,205	AB_2563932	1:125
MHCII AF700	BioLegend	Rat	Monoclonal	M5/114.15.2	107,621	AB_3208338	1:50
CD45 BV510	BioLegend	Rat	Monoclonal	30‐F11	103,137	AB_2563061	1:125
CD4 BV605	BioLegend	Rat	Monoclonal	RM4‐5	100,547	AB_2921040	1:125
Ly6C BV650	BioLegend	Rat	Monoclonal	HK1.4	128,049	AB_2800630	1:250
CD11c BV785	BioLegend	Hamster	Monoclonal	N418	117,335	AB_2565268	1:125
Ly6G BV421	BioLegend	Rat	Monoclonal	1A8	127,627	AB_2562567	1:125
CD49b BV480	BD Biosciences	Hamster	Monoclonal	HMalfa2	746,355	AB_2743674	1:250

### 2.11 Antigen Uptake and Processing Assays

Following lung harvest and LEC isolation, 1 × 10^6^ live cells (as determined by trypan blue) were resuspended in 180 μL of prewarmed serum‐free PromoCell Airway Epithelial Growth Medium. Following the addition of 20 μL of OVA‐AF647 (Invitrogen, USA) and DQ‐OVA (Invitrogen, USA)) at a final concentration of 10 μg/mL, cells were incubated at 37°C, 5% CO2 for 1 or 4 h. All samples were collected simultaneously for further antibody staining and flow cytometric analysis (as described above).

### 2.12 T‐Cell Proliferation Assays

Isolated LECs were placed in complete T‐cell media (RPMI +10% FBS + 1% L‐Glu + 100 U/mL Pen/Strep +1% MEM NEAA +1% Na‐Pyruvate +0.1% 2‐mercaptoethanol) (Gibco, USA) and SIINFEKL peptide (InvivoGen, USA) at 25 μg/mL for 4 h at 37°C 5% CO_2_. Meanwhile, inguinal, mesenteric, brachial, axillary, and superficial cervical lymph nodes (LNs) were harvested from OT‐I mice into cold complete T‐cell media. Lymph nodes were mashed and then processed using the lung harvest and processing protocol as described above. The CD8 T‐cell MojoSort (BioLegend, USA) protocol was followed for negative sorting of CD8+ T‐cells. Up to 5 × 10^7^ labelled cells were placed in the Miltenyi LS column on the OctaMACS magnet. Flowthrough containing CD8+ T cells was collected, and cells were stained with 1:5000 CFSE (Invitrogen, USA) according to the manufacturer's instructions. 1 × 10^5^ OT‐I CD8+ cells were added per 2 × 10^4^ LECs in a 96 round‐bottom well plate. Cells were co‐cultured in 200 μL complete T‐cell media for 72 h at 37°C 5% CO_2_, followed by antibody staining and flow cytometric analysis as described above (Figure S4). The proliferation index was calculated as the average number of cells that an initial cell became.

### 2.13 Data Presentation and Statistical Analysis

Data are represented as violin, split violin, scatter, or dot plots generated using “ggplot2” package [40] (release 3.18). “webr” package (release 0.1.5) was used to create donut charts, and “UpSetR” package (release 1.4.0) [41] was used to create an UpSet plot. Dark shaded areas represent the actual distribution of data, while lightly shaded areas represent the predicted probability density. Solid lines represent the median, while dashed lines represent the 0, 25th, 75th, and 100th percentile quartiles. R studio “rstatix” package [42] (release 0.7.2) was used for statistical analysis. The normality of the data was assessed using the Shapiro–Wilk test. For single‐variable analysis, a One‐Way ANOVA was employed, followed by the Holm post hoc test for multiple comparisons (where more than two groups were present). For dual‐variable analysis of multiple groups, a Two‐Way ANOVA was employed, followed by the Holm post hoc test for multiple comparisons. Statistical comparisons were added to plots using “ggpubr” package [42] (release 0.6.0).

## 3 Results

### 3.1 RSV Infection Results in Prolonged Epigenetic and Transcriptional Changes in LECs

**FIGURE 1:**
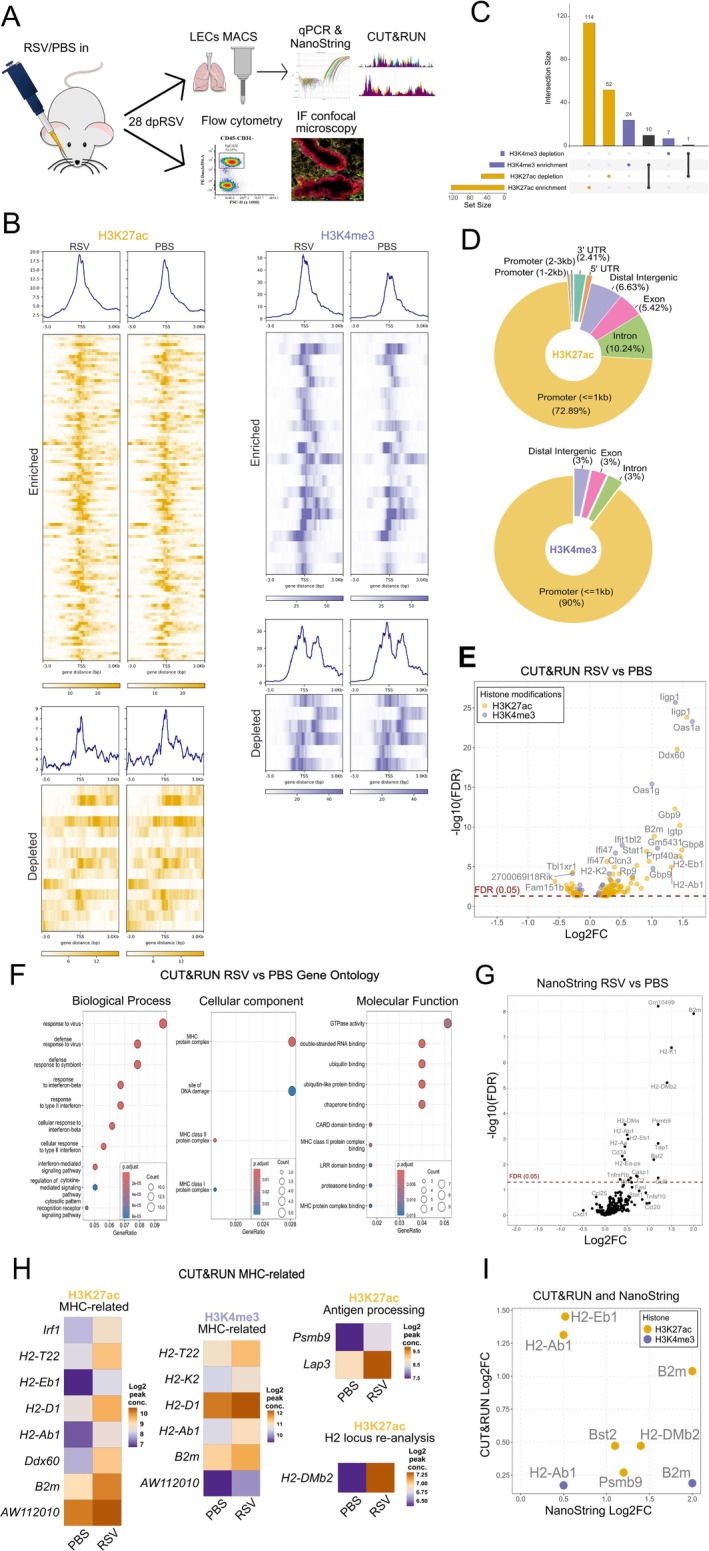
RSV causes prolonged epigenetic and transcriptional changes in LECs. (A) Experimental design. 6‐week‐old female BALB/c mice were administered RSV or PBS intranasally. After 28 days, mice were culled and lung epithelial cells were MACS‐sorted for NanoString or CUT&RUN analysis, or lungs were harvested for confocal immunofluorescence microscopy or flow cytometry analysis. (B) CUT&RUN peak profile heatmaps of significant differentially bound H3K4me3 and H3K27ac histone modifications—8 kb window. (C) UpSet plot of identified enriched and depleted DBS for H3K4me3 and H3K27ac (D) Pie charts of annotated genomic distribution of identified DBS for H3K4me3 and H2K27ac. (E) Volcano plot of genes associated with H3K4me3 and H3K27ac that were differentially bound in the RSV group as compared to the PBS group (FDR < 0.05, x axis shows a log2FC in enrichment/depletion for each histone modification, while y axis shows a ‐log10 FDR values). (F) Dot plot graph of gene ontology (GO) term enrichment for combined H3K4me3 and H3K27ac. GO terms were stratified based on one of three sub‐ontologies: Biological process (BP), cellular component (CC) and molecular function (MF). Surface area of each dot corresponds to the number of genes assigned to each term, while color levels correspond to the statistical significance of the discovered term. Gene Ratio on the x axis is a ratio of genes identified per GO term over the total number of genes assigned to that term. (G) Volcano plot of differentially expressed LEC genes 28dpRSV identified by NanoString nCounter mouse Immunology panel in the RSV group as compared to the PBS group. The x axis shows a log2FC in expression, while the y axis shows ‐log10 FDR values. (H) Heatmaps of relevant MHC or antigen processing‐related genes identified by CUT&RUN that were enriched in the RSV group. The murine H2 locus was re‐analyzed, resulting in identification of one additional, previously unreported target—*H2‐DMb2*. Log2 peak conc is a mean read concentration for the sample in each given group. (I) Scatter plot of all overlapping targets identified by both CUT&RUN and NanoString 28dpRSV. The y axis shows log2FC in histone enrichment, and the x axis shows the corresponding log2FC in gene expression, as identified by NanoString. CUT&RUN *n* = 4 per group. NanoString *n* = 6 per group. Each CUT&RUN sample is a pool of three mice, and each NanoString sample is a pool of two mice. Each experiment was performed twice.

To test our hypothesis that RSV infection results in prolonged changes in LECs, we employed a murine model of RSV infection and investigated epigenetic and transcriptomic changes long after viral clearance (Figure S6). Specifically, we focused on a timepoint 28 days post RSV infection (28dpRSV) given that RSV is cleared within 7 days, and therefore the virus had been cleared at least 3 weeks prior [43, 44, 45] (Figure 1A). First, to interrogate potential prolonged epigenetic changes, we performed CUT&RUN of histone 3 lysine 4 trimethylation (H3K4me3) and histone 3 lysine 27 acetylation (H3K27ac); both histone modifications are associated with transcriptional activation when present within the regulatory region of a gene [46, 47, 48]. We discovered 166 differentially bound sites (DBS) for histone modification H3K27ac and 31 dB for H3K4me3 in LECs 28dpRSV compared with controls (Figure 1B,C, Table S2). Importantly, the vast majority of identified DBS were associated with regulatory elements of corresponding genes; more than 72% of H3K27ac and 90% of H3K4me3 DBS were identified in promoter regions, suggesting that observed changes in histone modification enrichment may be functional (Figure 1D). Although both enriched and depleted DBS were identified, only enriched DBS exhibited strong statistical significance and magnitude of change (Figure 1E). Gene ontology term analysis identified viral and interferon responses, and several pathways related to major histocompatibility complexes (MHC) and antigen processing (Figure 1F).

Next, to determine the impact of RSV infection on the transcriptional profile of LECs, we used a NanoString Immunology panel. We discovered 19 differentially expressed genes; strikingly, eleven of those were associated with both classes of MHC and antigen processing (Figure 1G). This led us to reassess the CUT&RUN data in the context of MHC‐related genes. Of the 197 significant DBS, eight H3K27ac and six H3K4me3 were associated with MHC biology, while two H3K27ac DBS were associated with antigen processing (Figure 1H). Additionally, CUT&RUN analysis specifically within the H2 locus, where the majority of MHC‐related genes are located [49], revealed a single novel enriched H3K27ac DBS in the promoter of *H2‐DMb2*. There was also an overlap between prolonged epigenetic and transcriptomic changes. Six genes identified as differentially enriched by CUT&RUN were also differentially expressed (FDR < 0.05) in NanoString data (Figure 1I). Five of the genes are associated with MHC and antigen processing, while one gene, *Bst2*, is thought to be involved in antiviral responses [50]. Taken together, these data demonstrate that RSV infection results in prolonged transcriptional and epigenetic effects on LECs.

### 3.2 RSV Infection Alters the Expression of MHC and Associated Apparatus by LECs

**FIGURE 2:**
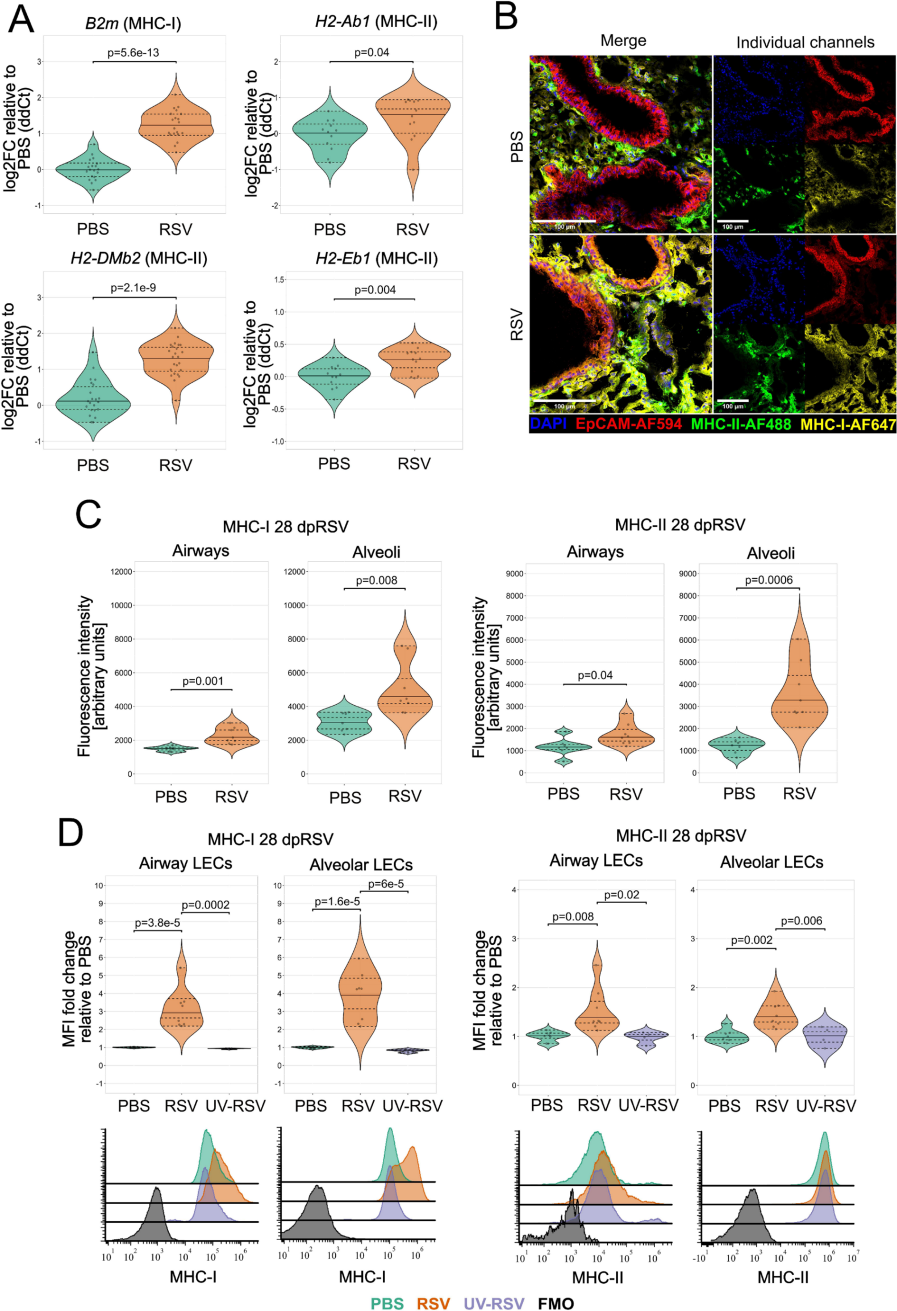
RSV infection results in prolonged upregulation of MHC‐I and MHC‐II on LECs. 6‐week‐old female BALB/c mice were administered RSV, PBS or UV‐RSV intranasally. 28dpRSV LECs were MACS‐sorted for qPCR, or lungs were processed for flow cytometry or confocal IF microscopy. (A) Duplex TaqMan qPCR validation of selected targets identified by CUT&RUN and NanoString, performed on MACS‐sorted LECs. *Oaz1* was used as endogenous control for *H2‐DMb2* and *H2‐Eb1* while *Gapdh* was used as endogenous control for *B2m* and *H2‐Ab1*. Data presented as log2FC relative to PBS. *N* = 12–24. Up to six samples used to generate data points in Figure 2A were also used to generate NanoString data in Figure 1G. (B) Representative confocal IF micrographs of MHC distribution in sucrose‐dehydrated 7 μm‐thick OCT‐embedded murine lung 28dpRSV. 40× magnification, white scale bar—100 μm. Blue DAPI—nuclei, red—EpCAM‐AF594, green—MHC‐II‐AF488, yellow—MHC‐I‐AF647. (C) Quantification of absolute fluorescence intensity (MHC‐I and MHC‐II) in 20× magnification micrographs in airways and alveoli based on EpCAM expression via automated StarDist segmentation. *N* = 7–8. (D) Relative flow cytometry quantification of MHC‐I and MHC‐II expression 28dpRSV stratified based on EpCAM expression corresponding to alveoli and airways. An additional UV‐RSV control group was included. Representative histograms are included in the bottom panel. *N* = 4–8. Each data point is an individual mouse. Each experiment was repeated at least twice. Statistical significance was determined by performing One‐Way ANOVA with Holm post hoc test.

Following the identification of several differentially expressed and histone‐modified MHC‐related genes, we validated the upregulation of several representative genes by qPCR (Figure 2A). We further used confocal microscopy to validate MHC upregulation at the protein level (Figure 2B,C), finding higher levels of both MHC‐I and MHC‐II in the airway and alveolar compartments 28dpRSV. Finally, MHC expression was assessed at single‐cell level by flow cytometry, which confirmed that CD45‐CD31‐EpCAM^high^ airway LECs and CD45‐CD31‐EpCAM^low^ alveolar LECs (Figure S5) had high expression of both MHC‐I and MHC‐II at 28dpRSV compared with PBS and UV‐RSV controls (Figure 2D), indicating that persistent MHC upregulation requires RSV infection and not just RSV antigen. Considering this, we investigated whether the magnitude of MHC expression correlates with infection severity by correlating weight change at 6 dpRSV (peak infection severity [45, 51]) with MHC expression increases 28dpRSV (Figure S7). Using Pearson correlation coefficient, we found that increases in the expression of MHC‐I in both lung compartments and of MHC‐II in the airways strongly correlate with infection severity. Considering the importance of co‐stimulatory molecules in initiating antigen presentation, we also assessed the expression of CD40, CD86, CD80, and OX40L. While little‐to‐no changes were observed for CD40 and CD86A, CD80 (Figure S8A) and OX40L were at or below the levels of detection (Figure S8B).

Thus, these data show that MHC expression is increased long after RSV infection is cleared.

### 3.3 Enhanced in Vivo Responsiveness to LPS by RSV‐Experienced LECs

**FIGURE 3:**
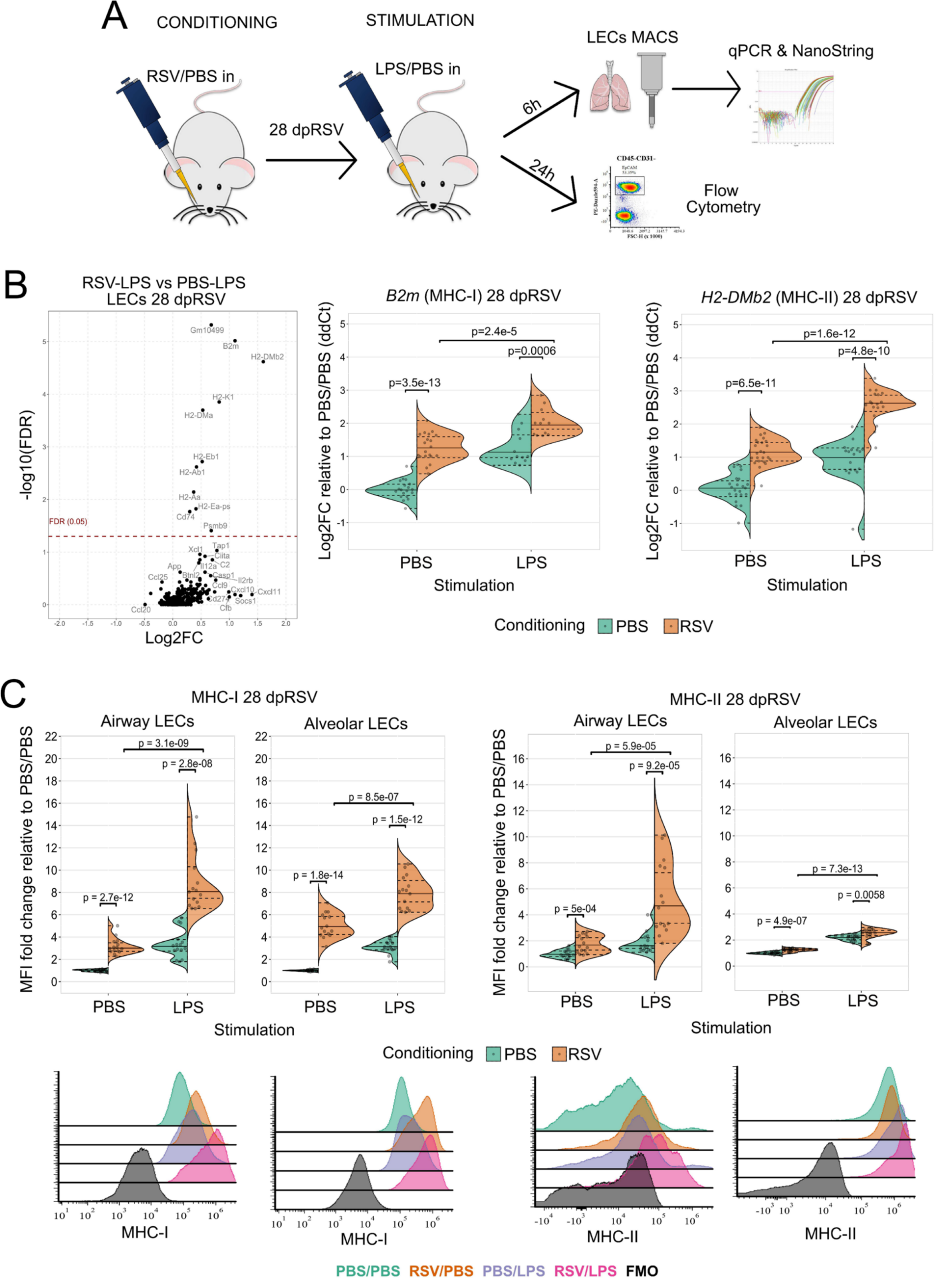
Intranasal LPS administration 28dpRSV results in an enhanced expression of MHC‐I and MHC‐II on LECs. (A) 28 days after administering RSV or PBS to 6‐week‐old female BALB/c mice 10 μg of LPS or PBS were administered intranasally. Either 6 h or 24 h later mice were culled for transcriptomic or flow cytometry analysis, respectively. (B) Left panel—volcano plot of differentially expressed LEC genes 28dpRSV identified by NanoString nCounter mouse Immunology panel in RSV‐LPS groups as compared to PBS‐LPS group. X axis shows a log2FC in expression, while y axis shows a ‐log10 FDR values. NanoString *n* = 6 per group, each sample is a pool of two mice, two independent experiments. Centre and right panels—representative MHC‐I and MHC‐II‐related targets validated using duplex TaqMan qPCR. *Oaz1* was used as endogenous control for *H2‐DMb2* while *Gapdh* was used as endogenous control for *B2m*. Treatment on day 0 (conditioning) is color coded, PBS—green and RSV—orange. Treatment on day 28 (stimulation with PBS or LPS) is labelled on x axis. *N* = 12–24 per group, four independent experiments. Up to six samples used to generate qPCR data points were also used to generate NanoString data in the same figure. (**C**) Relative flow cytometry quantification of MHC‐I and MHC‐II expression on LECs 28dpRSV stratified based on EpCAM expression which corresponds to alveolar and airway spaces. Representative histograms are included in the bottom panel. Treatment on day 0 (conditioning) is color coded, PBS—green and RSV—orange. Treatment on day 28 (stimulation with PBS or LPS) is labelled on x axis. *N* = 15 per group, four independent experiments. Two‐Way ANOVA with Holm post hoc test was performed to determine statistical significance.

Considering the prolonged epigenetic changes observed following RSV infection, we investigated if LEC responses to immunogens are altered. Lipopolysaccharide (LPS) has well‐described effects on LECs [52, 53, 54]; triggering proinflammatory transcriptional programs through TLR4/CD14/MD‐2 receptor signaling, similar to RSV F protein [55], and it is a major component of house dust mite (HDM) [56], a major respiratory allergen. LPS (or PBS) was therefore given intranasally (stimulation) to mice 28 days after RSV or PBS administration (conditioning), followed by transcriptome and protein analysis of LECs (Figure 3A).

Following 6 h in vivo LPS or PBS stimulation, the transcriptional response of isolated LECs was assessed using NanoString (Figure 3B). Virtually all significantly upregulated genes (RSV‐LPS vs. PBS‐LPS) were related to MHC biology, and the increased expression of *B2m* (MHC‐I) and *H2‐DMb2* (MHC‐II) was validated by qPCR (Figure 3B). Next, we used flow cytometry to assess the expression of MHC molecules on LECs 24 h after LPS administration, at 28dpRSV. Corroborating the qPCR data, RSV conditioning enhanced the response to LPS, with increased MHC‐I and MHC‐II expression in both airway and alveolar LECs (Figure 3C). Given that TLR4 can be upregulated on LECs following RSV infection [57] we assessed its expression using flow cytometry at 28 dpRSV and found no differences compared to control (Figure S9).

### 3.4 LECs Show Augmented Antigen Uptake, Processing, and Presentation 28dpRSV

**FIGURE 4:**
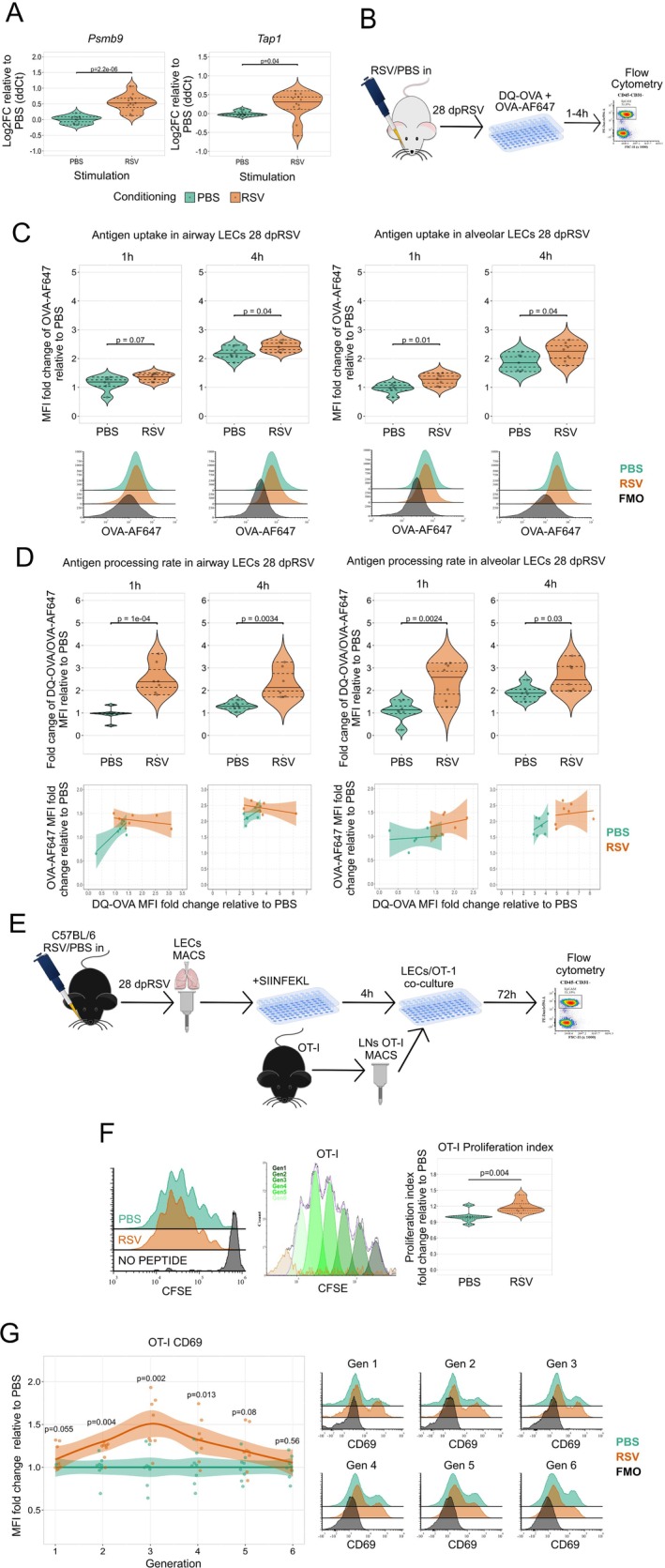
RSV infection results in enhanced LECs antigen uptake, processing and presentation. (A) Duplex TaqMan qPCR of *Psmb9* and *Tap1* with *Oaz1* as endogenous control in MACS‐sorted LECs 28dpRSV. (B) Experimental design for antigen uptake and antigen processing assessment. Mice were administered RSV or PBS intranasally and then culled 28 days later. Lung single cell suspension was placed in serum‐free media with DQ‐OVA and OVA‐AF647. After 1 or 4 h cells were collected and stained for flow cytometry analysis. (C) Relative flow cytometry quantification of antigen uptake as determined by intracellular OVA‐AF647 signal in LECs stratified based on alveolar and airway spaces. Representative histograms in the panel on the bottom. (D) Relative flow cytometry quantification of antigen processing as determined by a ratio of intracellular DQ‐OVA over OVA‐AF647 signal in LECs stratified based on alveolar and airway spaces. DQ‐OVA/OVA‐AF647 bivariate plots in the panel at the bottom, each data point corresponds to MFI fold change ratio of DQ‐OVA/OVA‐AF647 in each mouse (E) Experimental design for assessment of antigen presentation. C57BL/6 mice were administered RSV or PBS intranasally. 28 days later mice were culled and LECs were MACS‐sorted. Sorted LECs were placed in an in vitro culture with SIINFEKL OVA peptide for 4 h. In the meantime, lymph nodes were harvested from OT‐I mice and OT‐I CD8 T‐cells were MACS sorted. OT‐I cells were labelled with proliferation dye CFSE. LECs and OT‐I cells were then co‐cultured for 72 h, followed by flow cytometry analysis (F). Relative flow cytometry analysis of OT‐I proliferation using FCS express 7 proliferation index. Left panel—representative histograms, middle panel—automated identification of OT‐I generations, right panel—relative OT‐I proliferation index. *N* = 8 per experimental group, two independent experiments. (G) Relative flow cytometry analysis of CD69 MFI in OT‐I cells across six generations 72 h after co‐culture start with SIINFEKL‐fed LECs. *N* = 8 per experimental group, two independent experiments. PBS—green and RSV—orange. One‐Way ANOVA with Holm post hoc test was performed to determine statistical significance.

Across CUT&RUN and NanoString datasets, we identified several targets associated with antigen processing and trafficking at 28dpRSV, namely *Psmb9*, *Lap3*, *Tap1*, and *Xcl1*. The upregulation of *Psmb9 (*20S immunoproteasome subunit) and *Tap1* (transporter molecule responsible for trafficking processed antigens [58]) was further validated by qPCR 28dpRSV (Figure 4A). This led us to hypothesize that either the antigen uptake rate and/or the antigen processing rate in LECs may be altered 28dpRSV.

To test this hypothesis, we infected or mock infected mice, followed by isolation and culture of LECs 28dpRSV in the presence of fluorescent ovalbumin (OVA)‐AF647, used to indicate antigen uptake, and BODIPY FL dye‐containing DQ‐OVA (Figure 4B) which fluoresces proportionally to its proteolytic cleavage [59]. Thus, the ratio of DQ‐OVA and OVA‐AF647 reflects the rate of antigen processing while controlling for potential differences in antigen uptake. First, we assessed the ability of LECs to take up OVA‐AF647 antigen over 1 or 4 h, with data stratified to alveolar and airway LECs (Figure 4C). Increased antigen uptake was observed after RSV conditioning across both epithelial subsets and timepoints. Similarly, we assessed antigen processing rates in alveolar and airway LECs after 1 or 4 h (Figure 4D). Again, we observed that the antigen processing rate is elevated 28dpRSV across both cell types.

Next, to test if prolonged upregulation of MHC molecules results in enhanced antigen presentation, we measured the proliferation of transgenic OVA‐specific OT mouse T cells in response to OVA‐peptide presented by LECs. Due to MHC restriction [60] and the C57BL/6J genetic background of OT mice [60] we needed to test if the effects of RSV infection are recapitulated in C57BL/6J LECs. Similar to BALB/c mice, the expression of MHC‐I in C57BL/6 mice was highly upregulated 28 dpRSV; however, no prolonged change in expression of MHC‐II was observed (Figure S10).

Twenty Eight days after the inoculation of C57BL/6J mice with RSV, we harvested LECs and cultured them with or without OVA peptide recognized by OT‐I CD8 T‐cells (SIINFEKL). After 4 h, LECs were washed and put in a co‐culture with OT‐I cells labeled with CFSE proliferation dye (Figure 4E). After 72 h, OT‐I cells proliferated more when exposed to LECs that had previously experienced RSV (Figure 4F). Additionally, CD69, a T‐cell activation and T‐cell tissue residency marker, was significantly upregulated on initial generations of proliferating OT‐I cells in the RSV group (Figure 4G).

## 4 Discussion

Here, we demonstrate that RSV infection results in prolonged epigenetic changes in LECs, including in regulatory elements of multiple genes related to MHC molecules and antigen processing. Furthermore, we demonstrate an increased expression of MHC‐I and MHC‐II at transcriptional and protein levels in LECs 28dpRSV. The expression of both MHC classes in LECs can be further enhanced upon subsequent exposure to LPS. These epigenetic, transcriptomic, and protein changes appear functional, as we observe that 28dpRSV LECs display elevated antigen uptake and processing. Importantly, these changes are associated with an increased propensity for T‐cells to proliferate and become activated when antigen is presented by LECs that experienced RSV infection 28 days earlier.

Our findings have a twofold significance. On one hand, increased expression of MHC‐I following RSV may enhance protection against subsequent intracellular pathogens. On the other hand, the enhanced expression of MHC‐II may lead to increased presentation of extracellular antigens, including allergens, to CD4 T‐cells.

Mounting evidence suggests that the severity of influenza infection is reduced if mice are infected with RSV 3–4 weeks prior, as demonstrated by reduced morbidity, mortality, lung pathology, and immune cell infiltration [8, 61]. This phenomenon was shown to be independent of antibody‐mediated protection or cross‐protecting lung‐resident memory T‐cells (T_RM_) [62]. Here, we suggest that such protection could be a result of prolonged enhancement of antigen presentation by LECs. The importance of MHC antigen presentation during viral infection is underscored by the fact that many viruses, including SARS‐CoV‐2, influenza virus, cytomegalovirus, Epstein–Barr virus, HIV, rotavirus, and hepatitis B virus, actively interfere with MHC‐I expression, antigen loading, and presentation, including in epithelial cells [63, 64, 65, 66, 67, 68, 69]. In contrast, RSV infection upregulates MHC expression, as shown here in vivo in mice, as well as by other researchers in human lung epithelial cell lines [70, 71]. Interestingly, rhinovirus, like RSV, is associated with allergic airway disease development and exacerbation [72, 73], and also upregulates MHC in respiratory epithelial cells [74].

While enhanced antigen presentation by LECs may contribute to allergic asthma exacerbation, it is unlikely that heightened LEC allergen presentation alone will lead to the development of allergic airway disease. Rather, other factors like genetic predisposition (e.g., MHC alleles) or environmental factors (e.g., environmental LPS) contribute to the development of allergic airway disease. HLA genes are the most polymorphic genes found in humans [75], with more than 35,000 alleles identified as of 2023 [76]. It is therefore likely that only specific combinations of HLA alleles may increase the risk of respiratory allergy development, particularly considering that different MHC alleles have varying affinity for different peptides [77]. Indeed, it has been shown that different class II HLA genes have an impact on the course of asthma development [78] with HLA‐DQ identified as an asthma risk allele [75, 79, 80].

However, considering the increase in MHC levels following RSV infection and their further elevation after LPS exposure, it is conceivable that respiratory viral infection in the presence of environmental LPS may result in MHC‐mediated enhanced allergen presentation and thus contribute to allergic airway disease exacerbation in susceptible individuals. Multiple studies utilizing various experimental systems show that LPS exposure can result in exacerbation of allergic airway disease [81, 82, 83], while another study demonstrated that repeated LPS administration 35–41 days after RSV infection results in an aggravated inflammatory response and airway hyper‐responsiveness [84]. Nevertheless, in light of a report that lymph nodes are not essential for clonal expansion of allergen‐specific CD4 T‐cells in the murine lung [85], it is conceivable that prolonged MHC upregulation on LECs might suffice for the expansion of allergen‐specific lung‐resident T‐cell (T_RM_) populations. This is further supported by our observation that antigen presentation to OT‐I cells by LECs 28dpRSV leads to the upregulation of CD69, a marker of T_RM_ cells in the lung [86]. Consequently, following repeated allergen exposure, T_RM_ cells in conjunction with LECs alone could contribute to the development of allergic airway disease.

While MHC‐I and MHC‐II upregulation is generally driven by IFNγ [87, 88, 89], MHC‐I upregulation following RSV has been demonstrated to be primarily type I interferon (IFN‐I) mediated [71, 90]. Nevertheless, it is unlikely that IFNs alone are responsible for the observed phenotype. While C57BL/6 mice prototypically exhibit type 1 innate immune responses (T1), BALB/c mice tend to exhibit a type 2 innate immune response (T2) [91]. We observed that RSV infection upregulates MHC‐II on LECs in BALB/c mice but not in C57BL/6 mice, suggesting that while general antiviral responses may be responsible for the upregulation of MHC‐I, a T2 cytokine response may be responsible for the upregulation of MHC‐II. This notion is further reinforced considering that T2 cytokines like IL‐13 or IL‐33 correlate with RSV disease severity [45, 92], which in our model correlates with prolonged expression levels of MHC. Similarly, several T2 cytokines, including IL‐2, IL‐4, or IL‐13, induce MHC‐II expression [93, 94].

In summary, using a mouse model we have shown that RSV infection results in prolonged epigenetic and transcriptomic changes in lung epithelial cells that result in increased antigen presentation. Our findings increase understanding of epithelial cell memory after infection and could explain severity modulation of subsequent LRTI as well as the association between LRTI and the development of allergic airway disease.

## Conflicts of Interest

The authors declare no conflicts of interest.

## Supporting information


**Figure S1.** CD45‐CD31‐EpCAM+ LECs purity after MACS sorting. Following MACS sorting the purity of LECs was assessed using flow cytometry. Purity is evaluated as a % of CD45‐CD31‐EpCAM+ out of live cells.
Figure S2. QC of StarDist confocal immunofluorescent segmentation. EpCAM high airway regions are marked with green and EpCAM low alveolar regions are marked with yellow. Fields of view with incorrect segmentation were removed from analysis.
Figure S3. Representative flow cytometry gating for LECs analysis. Following debris exclusion, singlets are selected, and RBC are removed. Next live cells are selected followed by CD45‐CD31‐ selection. Within that population LECs are stratified to EpCAM low and EpCAM high.
Figure S4. Representative flow cytometry gating for analysis of CD8+ cells in OT1/LECs co‐culture after 72 h. Following debris exclusion, singlets are selected, and RBC are removed. Next, live cells are selected followed by CD45 + CD3+ selection. Within that population OT1 cells are identified as CD4‐CD8+.
Figure S5. Epithelial subset marker validation based on EpCAM expression. Based on confocal IF microscopy EpCAM is highly expressed in murine airway, while low expression is observed in alveoli. Similar EpCAM expression pattern is observed by flow cytometry following cold dispase digestion.
Figure S6. Characterization of BALB/c model of RSV infection. (A) Changes in mice weight in PBS (green) and RSV (orange) groups shown as % of initial weight. *N* = 20–42. Experiment was repeated at least four times. (B) Whole‐body plethysmography without a challenge following RSV administration on days 0–8. *N* = 4, single experiment. Whole body plethysmography was performed using a Buxco Max II preamplifier, Buxco bias flow regulator, Buxco mouse chambers, and FinePointe software. BALB/c mice were individually placed in measurement chambers, allowed to acclimatize for 5 min, and then base line lung function (without any challenge) was monitored over a 5‐min period on a daily basis for 8 days following RSV A2 administration. Enhanced pause (PenH) was calculated as an indicator of respiratory effort. (C) Amplification plots of duplex TaqMan qPCR of RSV L‐gene and Rpl37a endogenous control 3dpRSV and 28dpRSV. *N* = 3–4, single experiment. (D) Flow cytometric analysis of CD8/CD4 T‐cell ratio of mouse lung during peak T‐cell response. CD8 and CD4 T‐cells were identified as CD45 + CD3 + CD8 + CD4‐ and CD45 + CD3 + CD8‐CD4+, respectively. *N* = 4, single experiment. (E) H&E of lightly fixed, OCT‐inflated murine lungs 5dpRSV (peak inflammation), 28dpRSV or PBS control. Whole samples (half lung) were scanned using Zeiss Axioscan Z1 with 20× objective and three representative fields of view were selected. Black scale bar—60 μm. *N* = 6–8, two independent experiments. (F) Representative flow cytometry gating for analysis of major immune subsets in murine lung 28dpRSV. (G) Quantification of major immune cells subsets in control (PBS) lungs and 28dpRSV following liberase TL digestion. Absolute cell counts were back calculated based on a frequency of given cell population and total lung cell count. *N* = 4, single experiment.
Figure S7. Infection severity on D6 correlates with MHC expression on D28. Scatter plots of mouse weight change (in % vs. baseline) 6dpRSV (*y* axis) and MFI fold change of MHC‐I/MHC‐II expression (*x* axis) stratified based on anatomical niche (pink—airways, green—alveoli). The relationship strength between the variables was assessed by using a Pearson correlation coefficient.
Figure S8. Flow cytometric analysis of co‐stimulatory molecules on LECs 28dpRSV. (A) Relative flow cytometry quantification of CD40 and CD86 expression 28dpRSV stratified based on EpCAM expression corresponding to alveoli and airways and corresponding representative histograms. *N* = 11–15. Each data point is an individual mouse. Experiment was repeated at least thrice. Statistical significance was determined by performing One‐Way ANOVA with Holm post hoc test. (B) Representative histograms of CD80 and OX40L expression stratified based on EpCAM expression corresponding to alveoli and airways.
Figure S9. TLR4 abundance in LECs 28dpRSV as determined by flow cytometry analysis with representative histograms. *N* = 8 per group, two independent experiments.
Figure S10. Flow cytometry quantification of MHC‐I and MHC‐II expression in C57BL/6 mice 28dpRSV stratified to alveolar and airway spaces. Additional UV‐RSV control group was included. Representative histograms are included in the bottom panel. *N* = 4–8.
Table S1. Normfinder analysis of endogenous controls across experimental groups. The stability score is calculated based on experimental group differences and intragroup variances. The lower the stability score the more stable the endogenous control. GroupDif is a measure of differences between experimental groups, while GroupSD is a weighted average of SD in each group.
Table S2. List of all genes associated with statistically significant DBS across H3K4me3 and H3K27ac in RSV as compared to PBS 28dpRSV.

## Data Availability

The data that support the findings of this study are available from the corresponding author upon reasonable request.
