# Supplementary material for: Epithelial Memory After Respiratory Viral Infection in Mice Results in Prolonged Enhancement of Antigen Presentation
Source: Allergy. 2025 Aug 7;80(9):2501–18. doi: 10.1111/all.16683 (PMC12444829; doi:10.1111/all.16683)
Supplement: Supplementary file 1 — Figure S1. CD45‐CD31‐EpCAM+ LECs purity after MACS sorting. Following MACS sorting the purity of LECs was assessed using flow cytometry. Purity is evaluated as a % of CD45‐CD31‐EpCAM+ out of live cells. Figure S2. QC of StarDist confocal immunofluorescent segmentation. EpCAM high airway regions are marked with green and EpCAM low alveolar regions are marked with yellow. Fields of view with incorrect segmentation were removed from analysis. Figure S3. Representative flow cytometry gating for LECs analysis. Following debris exclusion, singlets are selected, and RBC are removed. Next live cells are selected followed by CD45‐CD31‐ selection. Within that population LECs are stratified to EpCAM low and EpCAM high. Figure S4. Representative flow cytometry gating for analysis of CD8+ cells in OT1/LECs co‐culture after 72 h. Following debris exclusion, singlets are selected, and RBC are removed. Next, live cells are selected followed by CD45 + CD3+ selection. Within that population OT1 cells are identified as CD4‐CD8+. Figure S5. Epithelial subset marker validation based on EpCAM expression. Based on confocal IF microscopy EpCAM is highly expressed in murine airway, while low expression is observed in alveoli. Similar EpCAM expression pattern is observed by flow cytometry following cold dispase digestion. Figure S6. Characterization of BALB/c model of RSV infection. (A) Changes in mice weight in PBS (green) and RSV (orange) groups shown as % of initial weight. N = 20–42. Experiment was repeated at least four times. (B) Whole‐body plethysmography without a challenge following RSV administration on days 0–8. N = 4, single experiment. Whole body plethysmography was performed using a Buxco Max II preamplifier, Buxco bias flow regulator, Buxco mouse chambers, and FinePointe software. BALB/c mice were individually placed in measurement chambers, allowed to acclimatize for 5 min, and then base line lung function (without any challenge) was monitored over a 5‐min period on a daily b [file ALL-80-2501-s001.pdf]

Figure S1

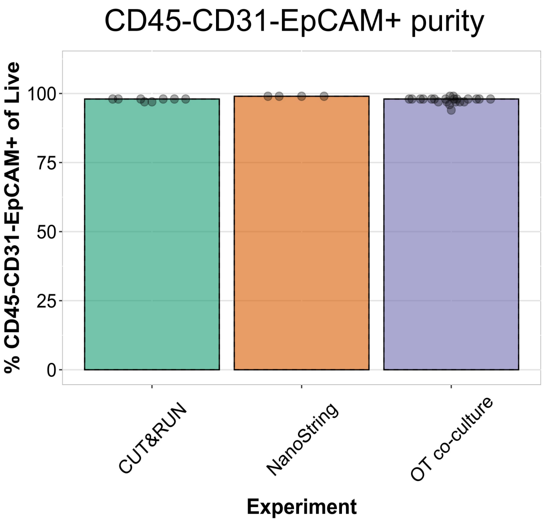

Figure S2

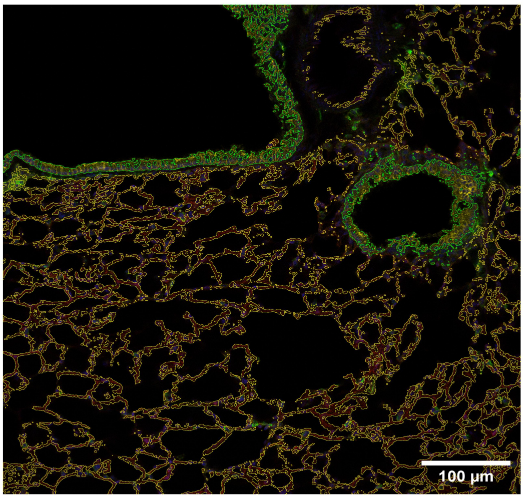

Figure S3

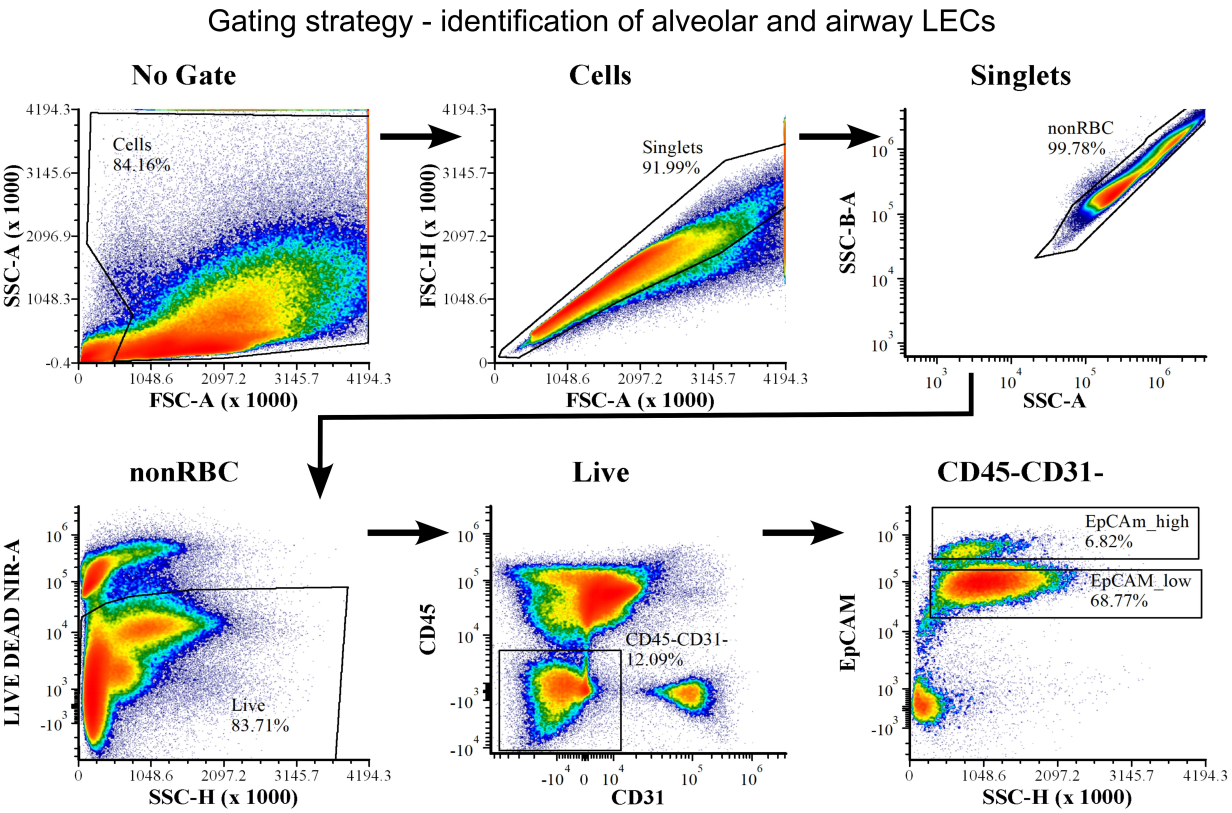

Figure S4

Gating strategy - OT-1/LECs co-culture 72h

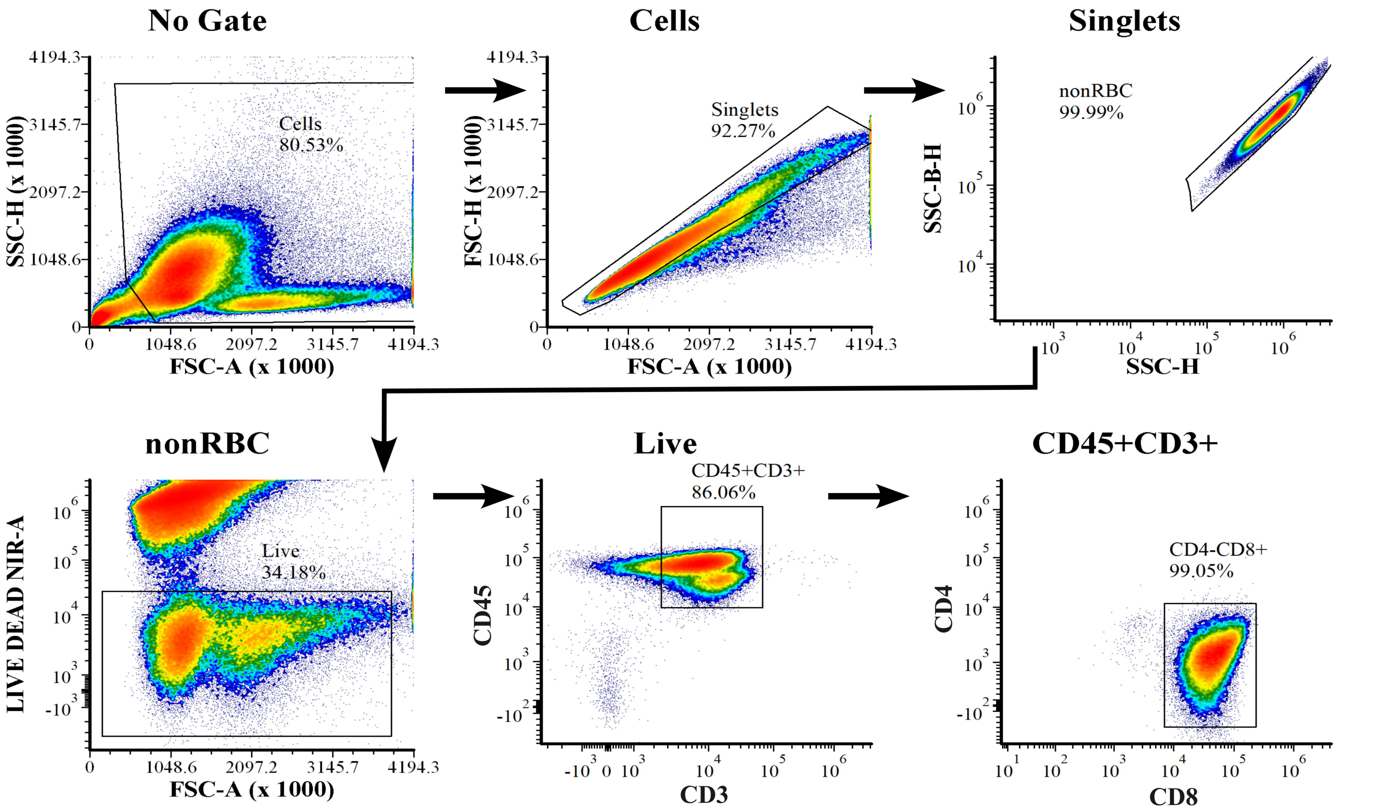

Figure S5

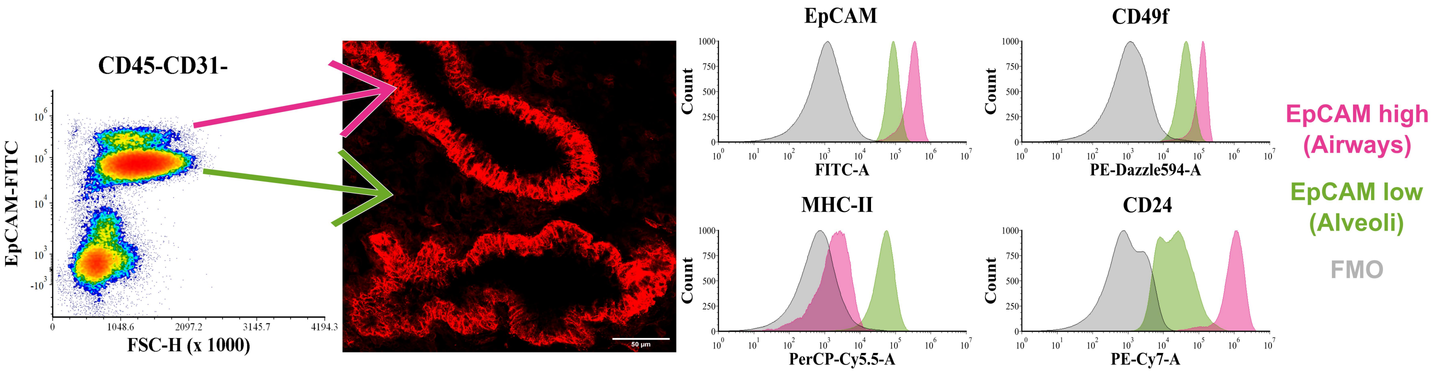

Figure S6

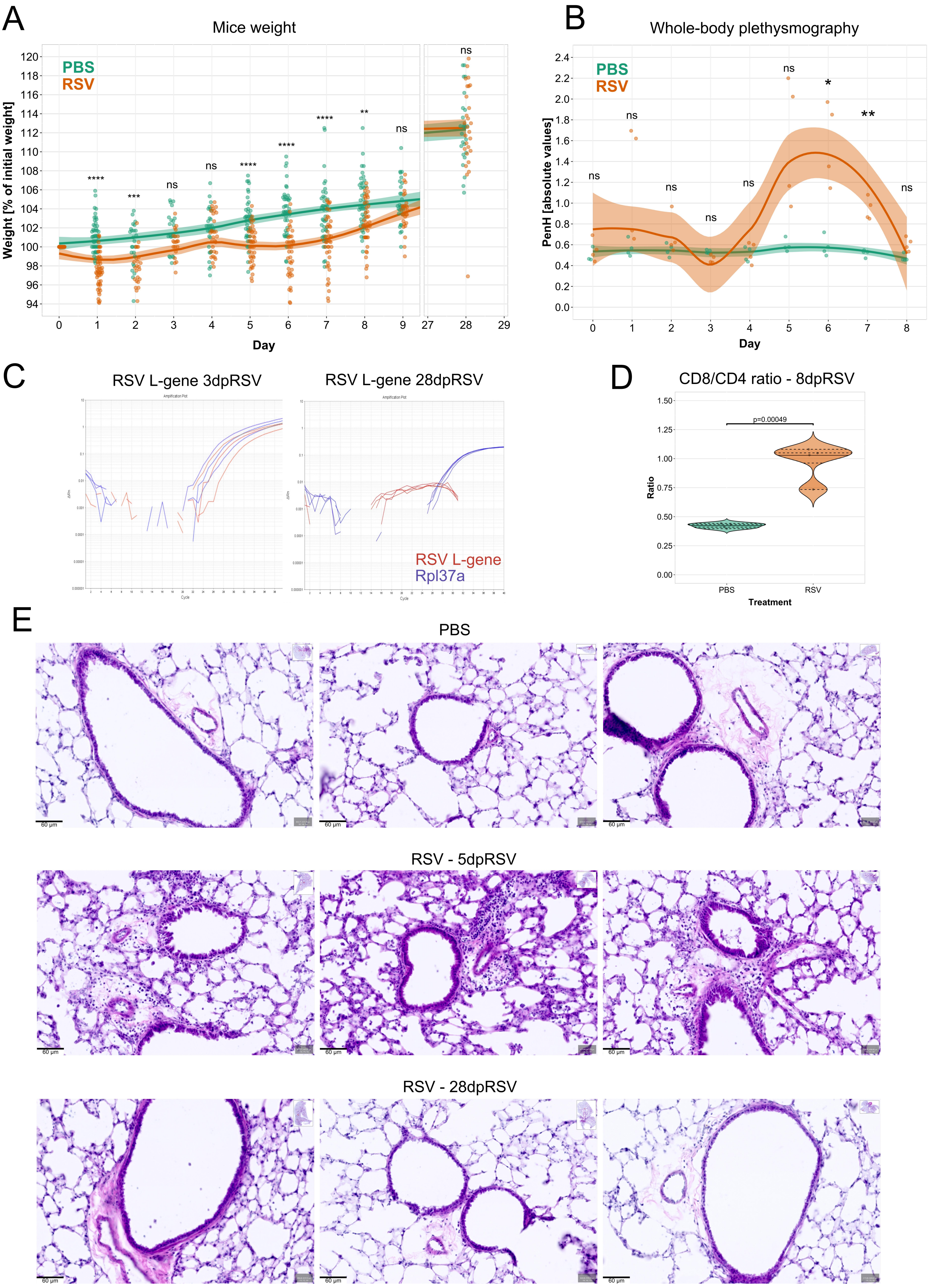

A
Mice weight
PBS
RSV
Weight [% of initial weight]
Day
B
Whole-body plethysmography
PenH [absolute values]
C
RSV L-gene 3dpRSV
RSV L-gene 28dpRSV
RSV L-gene
Rpl37a
D
CD8/CD4 ratio - 8dpRSV
p=0.00049
Ratio
Treatment
E
PBS
RSV - 5dpRSV
RSV - 28dpRSV
60 μm

F

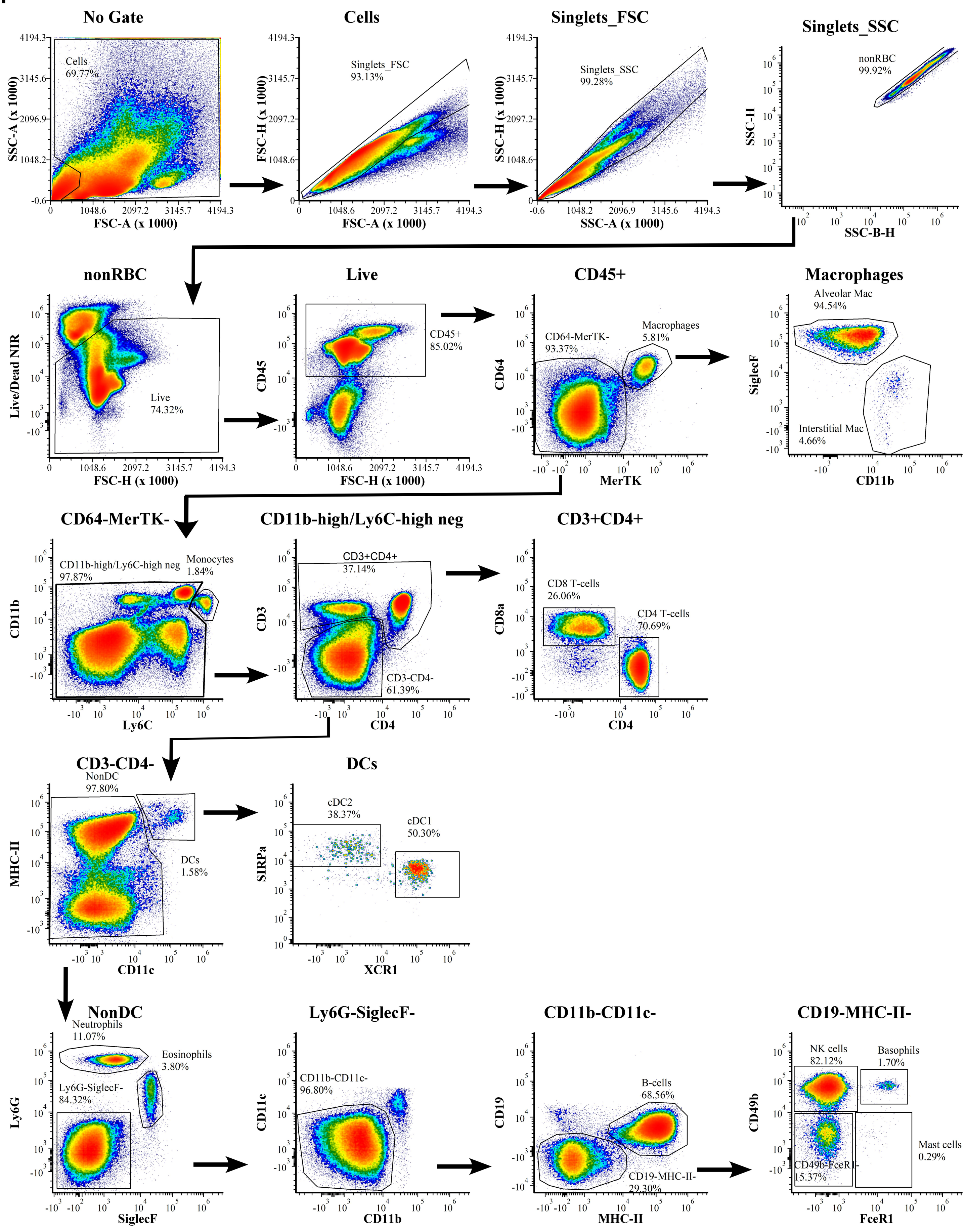
No Gate
Cells
69.77%
SSC-A (x 1000)
FSC-A (x 1000)
Cells
Singlets_FSC
93.13%
FSC-H (x 1000)
FSC-A (x 1000)
Singlets_FSC
Singlets_SSC
99.28%
FSC-H (x 1000)
SSC-A (x 1000)
Singlets_SSC
nonRBC
99.92%
SSC-H
SSC-B-H
nonRBC
Live
74.32%
Live/Dead NIR
FSC-H (x 1000)
Live
CD45+
85.02%
CD45
FSC-H (x 1000)
CD45+
CD64-MerTK-
93.37%
Macrophages
5.81%
CD64
MerTK
Macrophages
Alveolar Mac
94.54%
Interstitial Mac
4.66%
SiglecF
CD11b
CD64-MerTK-
CD11b-high/Ly6C-high neg
97.87%
Monocytes
1.84%
CD11b
Ly6C
CD11b-high/Ly6C-high neg
CD3+CD4+
37.14%
CD3-CD4-
61.39%
CD3
CD4
CD3+CD4+
CD8 T-cells
26.06%
CD4 T-cells
70.69%
CD8a
CD4
CD3-CD4-
NonDC
97.80%
DCs
1.58%
MHC-II
CD11c
DCs
cDC2
38.37%
cDC1
50.30%
SIRPa
XCR1
NonDC
Neutrophils
11.07%
Eosinophils
3.80%
Ly6G-SiglecF-
84.32%
Ly6G
SiglecF
Ly6G-SiglecF-
CD11b-CD11c-
96.80%
CD11c
CD11b
CD11b-CD11c-
B-cells
68.56%
CD19-MHC-II-
29.30%
CD19
MHC-II
CD19-MHC-II-
NK cells
82.12%
Basophils
1.70%
Mast cells
0.29%
CD49b-FceR1-
15.37%
CD49b
FceR1

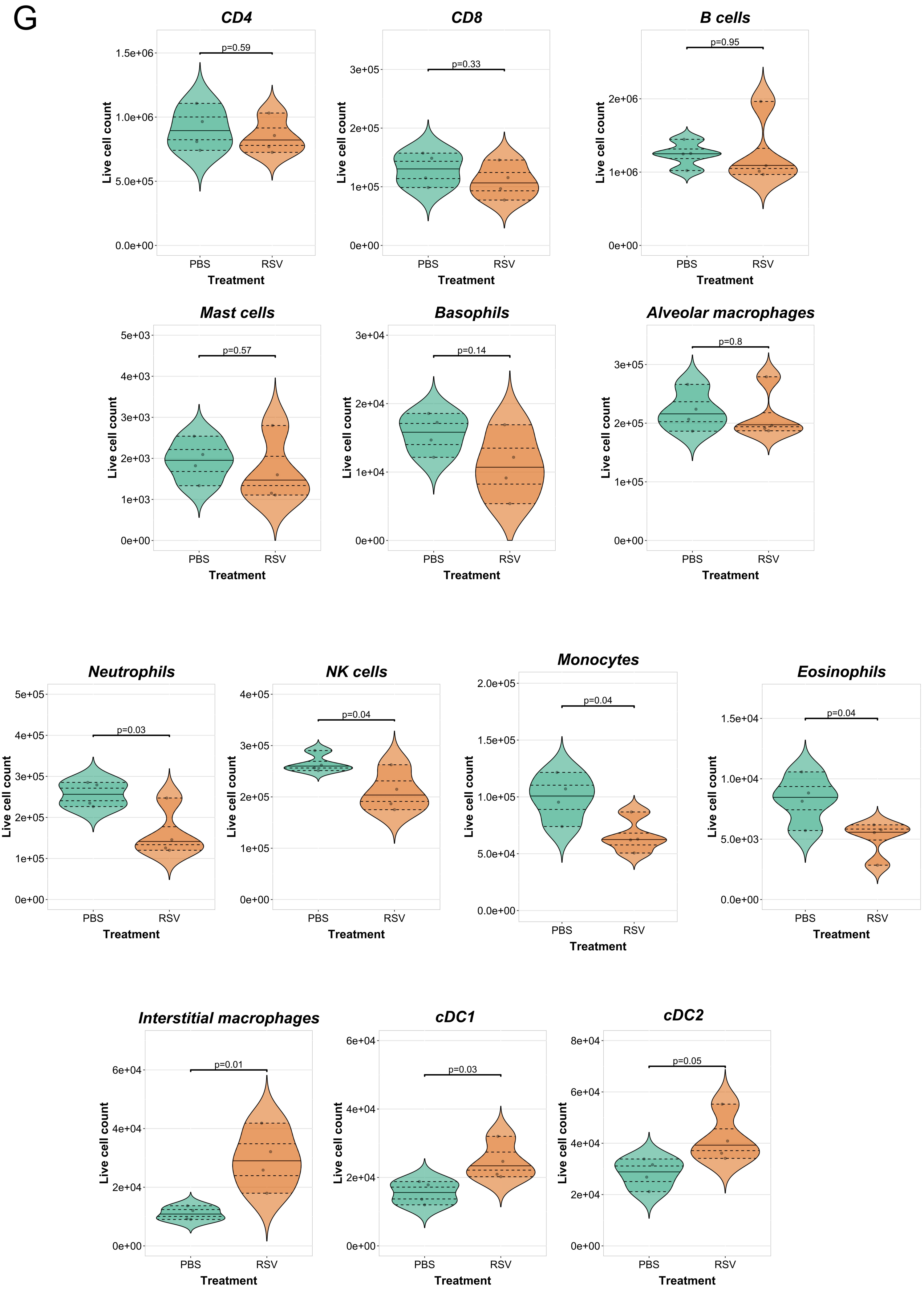
G
CD4
p=0.59
1.5e+06
1.0e+06
5.0e+05
0.0e+00
Live cell count
PBS
RSV
Treatment
CD8
p=0.33
3e+05
2e+05
1e+05
0e+00
B cells
p=0.95
2e+06
1e+06
0e+00
Mast cells
p=0.57
5e+03
4e+03
3e+03
2e+03
1e+03
0e+00
Basophils
p=0.14
3e+04
2e+04
1e+04
0e+00
Alveolar macrophages
p=0.8
3e+05
2e+05
1e+05
0e+00
Neutrophils
p=0.03
5e+05
4e+05
3e+05
2e+05
1e+05
0e+00
NK cells
p=0.04
4e+05
3e+05
2e+05
1e+05
0e+00
Monocytes
p=0.04
2.0e+05
1.5e+05
1.0e+05
5.0e+04
0.0e+00
Eosinophils
p=0.04
1.5e+04
1.0e+04
5.0e+03
0.0e+00
Interstitial macrophages
p=0.01
6e+04
4e+04
2e+04
0e+00
cDC1
p=0.03
6e+04
4e+04
2e+04
0e+00
cDC2
p=0.05
8e+04
6e+04
4e+04
2e+04
0e+00

Figure S7

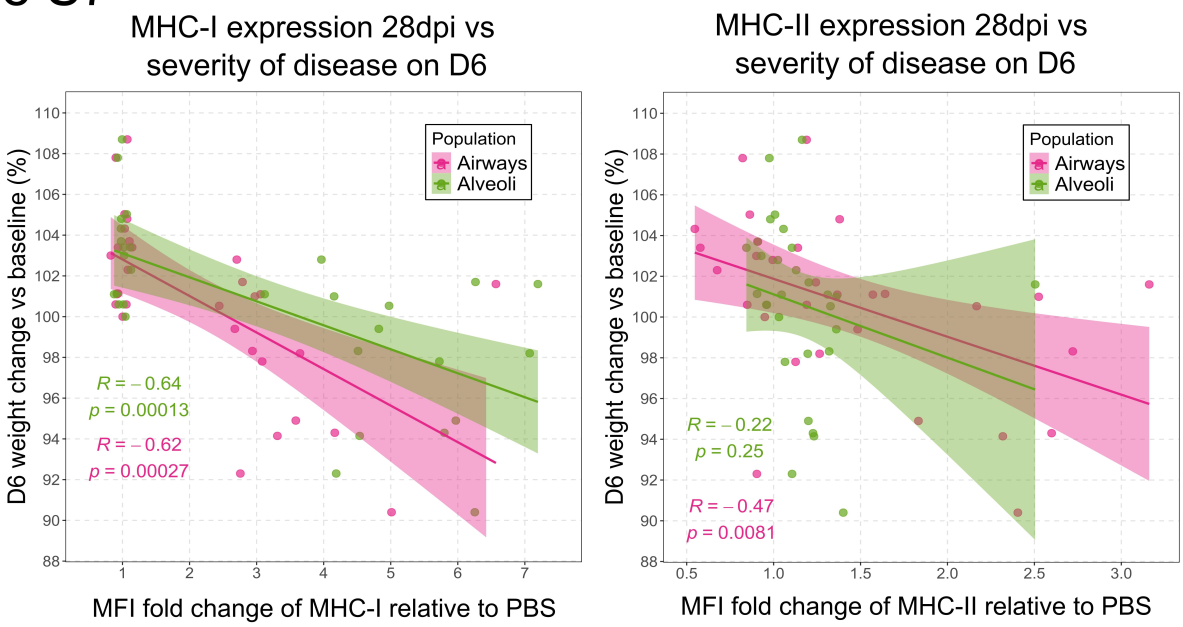

Figure S8

A

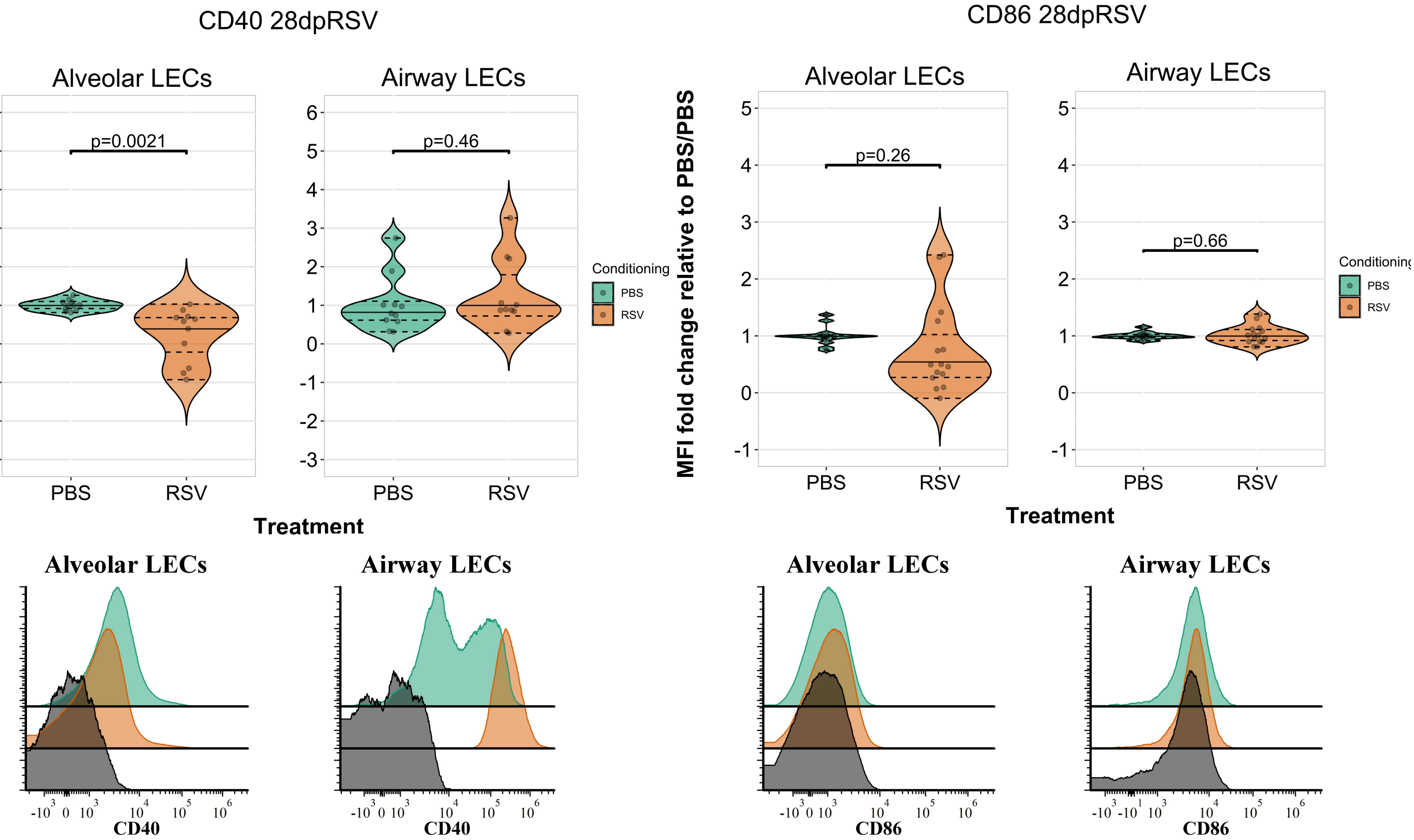

B

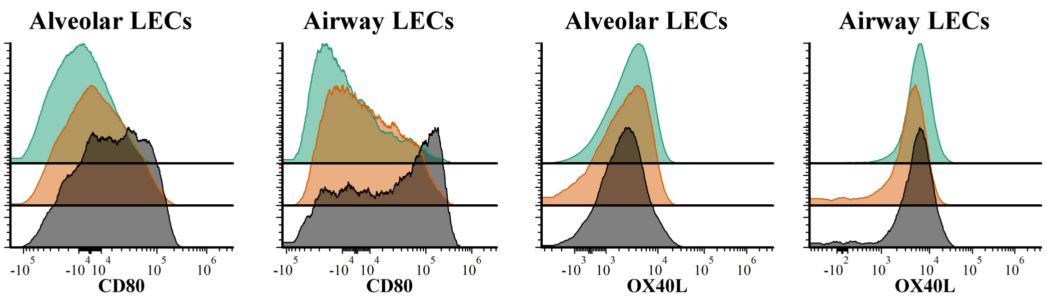

Figure S9

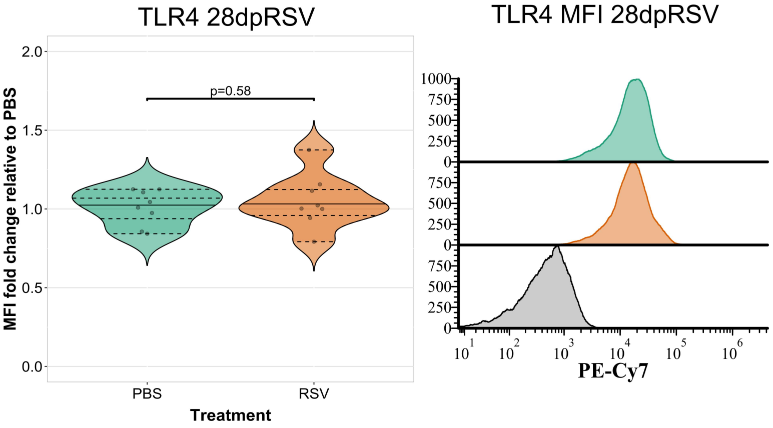

Figure S10

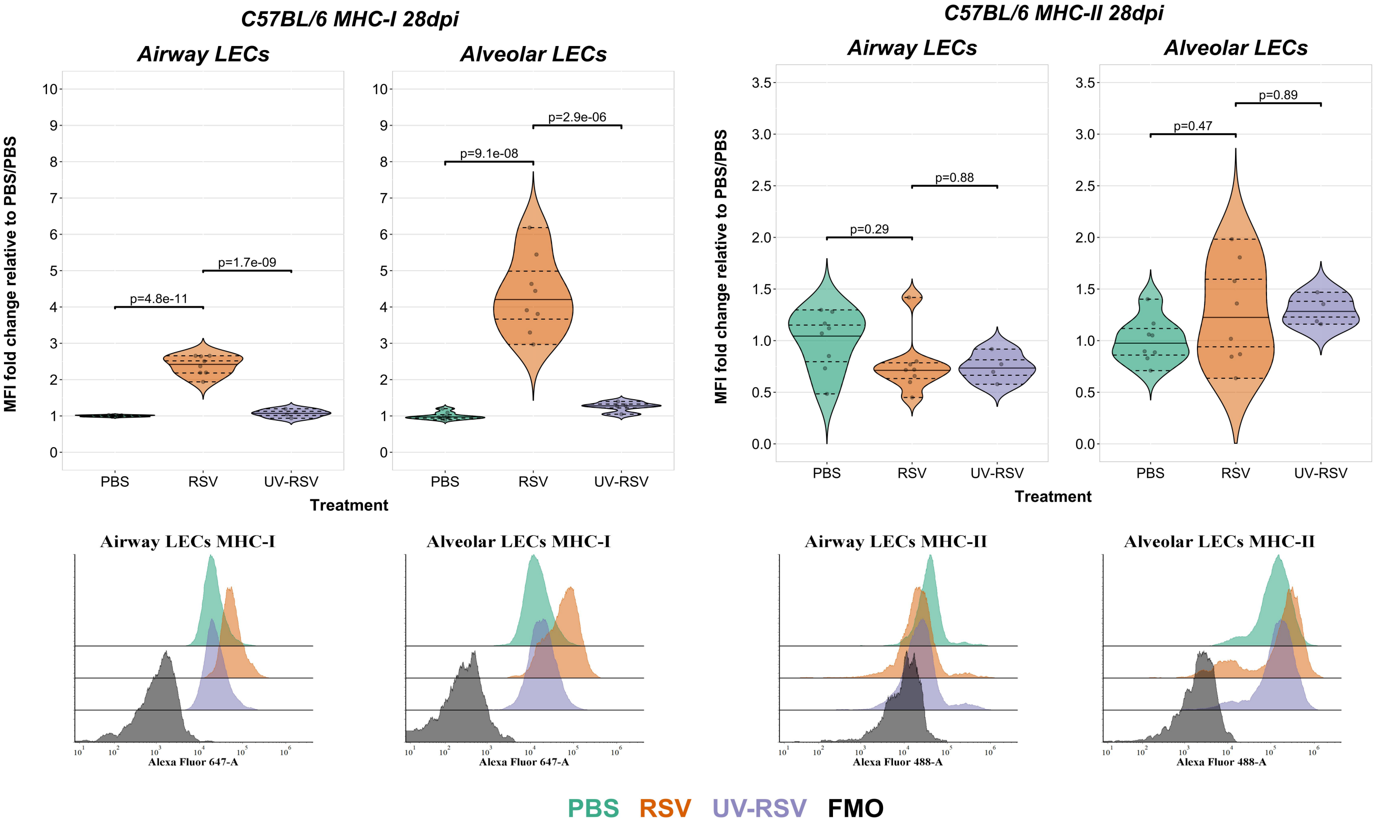

Table S1

Gene	GroupDif	GroupSD	**Stability score**
Oaz1	0.19	0.33	**0.13**
Gapdh	0.43	0.58	**0.21**
Rpl37a	0.71	0.65	**0.24**
18S	1.09	0.86	**0.28**

Table S2

Histone modification	SYMBOL	Conc_RSV	Conc_PBS	Fold	p.value	FDR	Genomic annotation	gene ID	ENSEMBL
H3K4me3	Iigp1	11.09	9.69	1.377542136	1.60E-30	2.06E-26	Promoter (<=1kb)	60440	ENSMUSG00000054072
H3K4me3	Oas1a	9.69	7.99	1.65259025	8.10E-28	5.20E-24	Promoter (<=1kb)	246730	ENSMUSG00000052776
H3K4me3	Oas1g	9.60	8.59	0.998866862	8.93E-20	3.82E-16	Promoter (<=1kb)	23960	ENSMUSG00000066861
H3K4me3	Ifit1bl2	10.43	9.88	0.519410604	6.19E-12	1.99E-08	Promoter (<=1kb)	112419	ENSMUSG00000067297
H3K4me3	Gm5431	10.17	9.04	1.08958445	1.87E-11	4.79E-08	Promoter (<=1kb)	432555	ENSMUSG00000058163
H3K4me3	Ifi47	11.99	11.55	0.411510731	8.88E-11	1.90E-07	Promoter (<=1kb)	15953	ENSMUSG00000078920
H3K4me3	Gbp9	9.72	8.66	1.016247832	9.27E-09	1.70E-05	Promoter (<=1kb)	236573	ENSMUSG00000029298
H3K4me3	H2-K2	10.82	10.46	0.308805978	5.14E-08	7.82E-05	Promoter (<=1kb)	630499	ENSMUSG00000121503
H3K4me3	2700069I18Rik	10.23	10.58	-0.285249807	5.49E-08	7.82E-05	Promoter (<=1kb)	72608	NA
H3K4me3	9330175E14Rik	9.88	9.12	0.687577858	1.43E-07	0.000183545	Promoter (<=1kb)	320377	NA
H3K4me3	Sorbs1	9.79	9.39	0.330893614	4.20E-07	0.00048974	Intron	20411	ENSMUSG00000025006
H3K4me3	Dhx58	9.77	9.22	0.458356247	5.62E-07	0.00060041	Promoter (<=1kb)	80861	ENSMUSG00000017830
H3K4me3	Spats2l	11.00	10.71	0.205803055	2.14E-06	0.00201797	Promoter (<=1kb)	67198	ENSMUSG00000038305
H3K4me3	Frzb	10.54	10.76	-0.163336089	2.34E-06	0.00201797	Promoter (<=1kb)	20378	ENSMUSG00000027004
H3K4me3	H2-D1	12.22	11.94	0.201098376	2.36E-06	0.00201797	Promoter (<=1kb)	14964	ENSMUSG00000073411
H3K4me3	H2-T22	11.39	10.87	0.385244993	3.01E-06	0.002409672	Promoter (<=1kb)	15039	ENSMUSG00000056116
H3K4me3	Tuba8	10.72	10.47	0.172048398	5.75E-06	0.004338839	Promoter (<=1kb)	53857	ENSMUSG00000030137
H3K4me3	Jarid2	10.11	10.42	-0.199222683	1.07E-05	0.007640145	Promoter (<=1kb)	16468	ENSMUSG00000038518
H3K4me3	Ptprd	10.72	10.97	-0.165996979	1.24E-05	0.007988958	Promoter (<=1kb)	19266	ENSMUSG00000028399
H3K4me3	Ciart	10.31	9.89	0.258762407	1.25E-05	0.007988958	Promoter (<=1kb)	229599	ENSMUSG00000038550
H3K4me3	Tab2	11.44	11.61	-0.128948083	1.46E-05	0.008938019	Promoter (<=1kb)	68652	ENSMUSG00000015755
H3K4me3	Ptprq	9.76	10.08	-0.183194781	2.86E-05	0.016649103	Distal Intergenic	237523	ENSMUSG00000035916
H3K4me3	Rcan1	11.03	10.82	0.140214634	3.46E-05	0.019317788	Promoter (<=1kb)	54720	ENSMUSG00000022951
H3K4me3	B2m	11.57	11.20	0.188822989	4.64E-05	0.024813953	Promoter (<=1kb)	12010	ENSMUSG00000060802
H3K4me3	Cald1	10.46	10.19	0.148853144	5.56E-05	0.028518044	Exon	109624	ENSMUSG00000029761
H3K4me3	Pcdh9	11.13	11.38	-0.144280672	6.58E-05	0.03247394	Promoter (<=1kb)	211712	ENSMUSG00000055421
H3K4me3	Oasl2	10.32	9.76	0.196252947	7.37E-05	0.033821296	Promoter (<=1kb)	23962	ENSMUSG00000029561
H3K4me3	AW112010	9.94	9.35	0.199918703	7.45E-05	0.033821296	Promoter (<=1kb)	107350	ENSMUSG00000075010
H3K4me3	H2-Ab1	10.96	10.55	0.172218535	7.65E-05	0.033821296	Promoter (<=1kb)	14961	ENSMUSG00000073421
H3K4me3	Ifi44	9.86	9.46	0.166103544	8.43E-05	0.036055844	Promoter (<=1kb)	99899	ENSMUSG00000028037
H3K4me3	Rexo1	11.20	11.00	0.125997305	9.62E-05	0.039794855	Promoter (<=1kb)	66932	ENSMUSG00000047417
H3K27ac	Iigp1	11.24	9.62	1.562787554	8.42E-29	1.48E-24	Promoter (<=1kb)	60440	ENSMUSG00000054072
H3K27ac	Ddx60	9.33	7.84	1.407686453	1.80E-24	1.58E-20	Promoter (<=1kb)	234311	ENSMUSG00000037921
H3K27ac	Gbp9	9.48	8.01	1.370888755	8.91E-17	5.23E-13	Promoter (<=1kb)	236573	ENSMUSG00000029298
H3K27ac	Igtp	9.16	7.57	1.449220429	1.42E-14	6.23E-11	Promoter (<=1kb)	16145	ENSMUSG00000078853
H3K27ac	B2m	10.13	9.02	1.038191433	4.22E-13	1.49E-09	Promoter (<=1kb)	12010	ENSMUSG00000060802
H3K27ac	Gbp8	8.52	6.85	1.481282945	2.70E-11	7.93E-08	Promoter (<=1kb)	76074	ENSMUSG00000034438
H3K27ac	Stat1	8.90	7.91	0.910862368	4.77E-11	1.20E-07	Promoter (<=1kb)	20846	ENSMUSG00000026104
H3K27ac	H2-Eb1	8.32	6.72	1.452174413	2.21E-10	4.87E-07	Promoter (<=1kb)	14969	ENSMUSG00000060586
H3K27ac	Prpf40a	9.56	8.50	0.952887182	1.23E-09	2.16E-06	Promoter (<=1kb)	56194	ENSMUSG00000061136
H3K27ac	Ifi47	12.12	11.80	0.271844774	1.38E-09	2.20E-06	Promoter (<=1kb)	15953	ENSMUSG00000078920
H3K27ac	Rp9	8.52	7.66	0.755081135	4.84E-09	7.10E-06	Promoter (<=1kb)	55934	ENSMUSG00000032239
H3K27ac	Clcn3	10.03	9.54	0.393629029	8.43E-09	1.14E-05	Promoter (<=1kb)	12725	ENSMUSG00000004319
H3K27ac	H2-Ab1	8.59	7.07	1.312938172	9.08E-09	1.14E-05	Promoter (<=1kb)	14961	ENSMUSG00000073421
H3K27ac	Tbl1xr1	10.59	10.93	-0.275526686	4.15E-08	4.87E-05	5' UTR	81004	ENSMUSG00000027630
H3K27ac	Spats2l	10.98	10.51	0.370146046	7.49E-08	8.25E-05	Promoter (<=1kb)	67198	ENSMUSG00000038305
H3K27ac	Ccng2	9.97	9.36	0.474725443	8.13E-08	8.42E-05	Promoter (<=1kb)	12452	ENSMUSG00000029385
H3K27ac	Brwd3	9.22	8.37	0.674553896	3.24E-07	0.000302857	Promoter (<=1kb)	382236	ENSMUSG00000063663
H3K27ac	AW112010	10.57	10.17	0.314035251	3.29E-07	0.000302857	Promoter (<=1kb)	107350	ENSMUSG00000075010
H3K27ac	H2-D1	9.65	8.54	0.918208757	3.56E-07	0.000302857	Promoter (<=1kb)	14964	ENSMUSG00000073411
H3K27ac	Zbp1	8.99	8.14	0.69132064	3.61E-07	0.000302857	Promoter (<=1kb)	58203	ENSMUSG00000027514
H3K27ac	Fam151b	8.31	9.06	-0.564270295	9.01E-07	0.000721485	3' UTR	73942	ENSMUSG00000034334
H3K27ac	Gm5431	10.02	9.54	0.353095569	9.74E-07	0.000737092	Promoter (<=1kb)	432555	ENSMUSG00000058163
H3K27ac	Mia	9.56	8.96	0.430899038	1.00E-06	0.000737092	Promoter (<=1kb)	12587	ENSMUSG00000089661
H3K27ac	Zxdc	9.20	9.61	-0.294318248	1.86E-06	0.001274843	3' UTR	80292	ENSMUSG00000034430
H3K27ac	Carf	8.44	7.53	0.658420834	1.88E-06	0.001274843	Promoter (<=1kb)	241066	ENSMUSG00000026017
H3K27ac	Hdgf	9.97	8.95	0.75499166	2.46E-06	0.001601168	Promoter (<=1kb)	15191	ENSMUSG00000004897
H3K27ac	Spag9	10.26	9.76	0.344176796	2.62E-06	0.00165044	Promoter (<=1kb)	70834	ENSMUSG00000020859
H3K27ac	Cap1	10.43	9.89	0.362499209	3.33E-06	0.002022642	Promoter (<=1kb)	12331	ENSMUSG00000028656
H3K27ac	Caprin1	9.86	9.26	0.396977602	3.64E-06	0.002137967	Promoter (<=1kb)	53872	ENSMUSG00000027184
H3K27ac	1700108F19Rik	8.21	8.77	-0.380037272	3.78E-06	0.002144844	Distal Intergenic	73272	ENSMUSG00000101009
H3K27ac	Gm12185	9.06	8.42	0.448034704	4.18E-06	0.002298405	Promoter (<=1kb)	620913	ENSMUSG00000048852
H3K27ac	Qars	9.17	8.40	0.508643967	5.71E-06	0.002981191	Promoter (<=1kb)	97541	ENSMUSG00000032604
H3K27ac	Erp29	9.31	8.83	0.328504888	5.90E-06	0.002981191	Promoter (<=1kb)	67397	ENSMUSG00000029616
H3K27ac	Klhl21	10.54	10.10	0.30274333	5.93E-06	0.002981191	Promoter (<=1kb)	242785	ENSMUSG00000073700
H3K27ac	Dnajc1	10.02	10.37	-0.255227412	6.41E-06	0.003134302	Intron	13418	ENSMUSG00000026740
H3K27ac	Tcta	10.63	10.29	0.254327877	7.07E-06	0.003363751	Promoter (<=1kb)	102791	ENSMUSG00000039461
H3K27ac	Xrcc1	9.49	8.70	0.497903077	7.57E-06	0.003488983	Promoter (<=1kb)	22594	ENSMUSG00000051768
H3K27ac	Wbp2	9.76	9.02	0.464559185	7.73E-06	0.003488983	Promoter (<=1kb)	22378	ENSMUSG00000034341

Table S2 cd.

Histone modification	SYMBOL	Conc_RSV	Conc_PBS	Fold	p.value	FDR	Genomic annotation	gene ID	ENSEMBL
H3K27ac	Mir6381	10.48	9.89	0.365865443	8.85E-06	0.003893946	Promoter (<=1kb)	102465200	ENSMUSG00000098871
H3K27ac	Abcc5	8.40	7.38	0.615806542	1.21E-05	0.005146703	Promoter (<=1kb)	27416	ENSMUSG00000022822
H3K27ac	Lacc1	8.31	8.82	-0.323654275	1.23E-05	0.005146703	Distal Intergenic	210808	ENSMUSG00000044350
H3K27ac	Il4i1b	9.37	7.96	0.827421003	1.34E-05	0.00548406	Promoter (<=1kb)	100328588	ENSMUSG00000074141
H3K27ac	Cdk17	9.97	10.42	-0.287634382	1.41E-05	0.005588339	Intron	237459	ENSMUSG00000020015
H3K27ac	Lnx2	10.00	9.46	0.33539185	1.43E-05	0.005588339	Promoter (<=1kb)	140887	ENSMUSG00000016520
H3K27ac	Yeats2	8.60	9.20	-0.369181587	1.57E-05	0.006019341	Exon	208146	ENSMUSG00000041215
H3K27ac	Cry2	11.78	11.31	0.301651847	1.80E-05	0.006731881	Promoter (<=1kb)	12953	ENSMUSG00000068742
H3K27ac	Angpt2	8.49	9.02	-0.327026473	1.90E-05	0.006925112	Intron	11601	ENSMUSG00000031465
H3K27ac	Zc3h4	11.51	11.24	0.205475406	1.93E-05	0.006925112	Promoter (<=1kb)	330474	ENSMUSG00000059273
H3K27ac	Gga1	9.84	8.97	0.491624414	2.03E-05	0.007132008	Promoter (<=1kb)	106039	ENSMUSG00000033128
H3K27ac	Slc35f3	8.49	9.10	-0.36201081	2.46E-05	0.008483181	Intron	210027	ENSMUSG00000057060
H3K27ac	Ilrun	10.08	9.73	0.249435652	2.53E-05	0.008577637	Promoter (<=1kb)	224647	ENSMUSG00000056692
H3K27ac	Snora33	8.82	8.01	0.439199343	2.74E-05	0.009089682	Promoter (<=1kb)	100529074	ENSMUSG00000070063
H3K27ac	Gm10575	10.77	10.13	0.360330583	3.14E-05	0.010232508	Promoter (<=1kb)	100126795	ENSMUSG00000073787
H3K27ac	Herc6	10.00	9.51	0.298955373	3.48E-05	0.011132232	Promoter (<=1kb)	67138	ENSMUSG00000029798
H3K27ac	Wfdc1	8.25	7.02	0.605547974	3.73E-05	0.011733442	Exon	67866	ENSMUSG00000023336
H3K27ac	Myo1c	10.39	9.66	0.385957503	3.97E-05	0.012258683	Promoter (<=1kb)	17913	ENSMUSG00000017774
H3K27ac	Glrx2	8.96	8.42	0.301778864	4.18E-05	0.012677013	Promoter (<=1kb)	69367	ENSMUSG00000018196
H3K27ac	Acd	8.23	7.46	0.397922322	4.44E-05	0.013049677	Promoter (<=1kb)	497652	ENSMUSG00000038000
H3K27ac	Mir92b	10.11	9.61	0.295272337	4.47E-05	0.013049677	Promoter (<=1kb)	100124470	ENSMUSG00000076255
H3K27ac	Paxx	10.19	9.76	0.268453517	4.52E-05	0.013049677	Promoter (<=1kb)	227622	ENSMUSG00000047617
H3K27ac	Bst2	9.11	8.16	0.471808744	4.66E-05	0.013248145	Promoter (<=1kb)	69550	ENSMUSG00000046718
H3K27ac	Zfp963	9.33	8.62	0.371133169	4.79E-05	0.013391464	Promoter (<=1kb)	620419	ENSMUSG00000092260
H3K27ac	Cct7	10.06	9.32	0.379180105	5.06E-05	0.013909359	Promoter (<=1kb)	12468	ENSMUSG00000030007
H3K27ac	Ankrd28	8.65	7.99	0.339192408	5.99E-05	0.01623362	Promoter (<=1kb)	105522	ENSMUSG00000014496
H3K27ac	Pcgf5	8.39	6.95	0.561697894	6.10E-05	0.016261777	Promoter (1-2kb)	76073	ENSMUSG00000024805
H3K27ac	Smarca5	9.80	9.06	0.364808958	6.26E-05	0.016457431	Promoter (<=1kb)	93762	ENSMUSG00000031715
H3K27ac	Lcorl	8.90	7.51	0.550675117	6.36E-05	0.016459782	Promoter (<=1kb)	209707	ENSMUSG00000015882
H3K27ac	Pvt1	9.22	9.70	-0.274800678	6.71E-05	0.017123695	Intron	19296	ENSMUSG00000097039
H3K27ac	Pigr	8.45	7.66	0.366913739	7.01E-05	0.017289083	Promoter (<=1kb)	18703	ENSMUSG00000026417
H3K27ac	Otub1	8.30	7.41	0.414440761	7.12E-05	0.017289083	Promoter (<=1kb)	107260	ENSMUSG00000024767
H3K27ac	Robo1	7.84	8.46	-0.320224971	7.15E-05	0.017289083	Intron	19876	ENSMUSG00000022883
H3K27ac	Adss	10.55	10.23	0.225757438	7.24E-05	0.017289083	Promoter (<=1kb)	11566	ENSMUSG00000015961
H3K27ac	Slc25a17	11.50	11.75	-0.188256378	7.27E-05	0.017289083	Promoter (<=1kb)	20524	ENSMUSG00000022404
H3K27ac	Dnaaf10	8.89	8.09	0.373186544	7.96E-05	0.018681239	Promoter (<=1kb)	103784	ENSMUSG00000078970
H3K27ac	Ppp2r2b	9.73	10.11	-0.240155285	8.41E-05	0.019165999	Intron	72930	ENSMUSG00000024500
H3K27ac	Nop16	9.05	8.40	0.318264948	8.49E-05	0.019165999	Promoter (<=1kb)	28126	ENSMUSG00000025869
H3K27ac	Chchd3	9.08	9.53	-0.263259707	8.85E-05	0.019720478	Intron	66075	ENSMUSG00000053768
H3K27ac	Cds2	10.07	9.23	0.37088334	9.63E-05	0.021199206	Promoter (<=1kb)	110911	ENSMUSG00000058793
H3K27ac	Hnrnpu	10.04	9.71	0.218300713	9.94E-05	0.021611282	Promoter (<=1kb)	51810	ENSMUSG00000039630
H3K27ac	Gbp7	8.68	7.83	0.368998042	0.000100747	0.021633562	Promoter (<=1kb)	229900	ENSMUSG00000040253
H3K27ac	Txlng	9.56	9.02	0.290581399	0.000103154	0.021883607	Promoter (<=1kb)	353170	ENSMUSG00000038344
H3K27ac	Gpatch2	9.22	9.58	-0.227964081	0.000106848	0.022322721	Intron	67769	ENSMUSG00000039210
H3K27ac	Pex5	9.52	9.13	0.239593628	0.000108447	0.022322721	Promoter (<=1kb)	19305	ENSMUSG00000005069
H3K27ac	Desi2	8.85	9.27	-0.250754675	0.000109027	0.022322721	5' UTR	78825	ENSMUSG00000026502
H3K27ac	Iqsec1	12.26	12.48	-0.172809951	0.000110792	0.022417225	Promoter (<=1kb)	232227	ENSMUSG00000034312
H3K27ac	Atpaf2	8.79	8.12	0.319741457	0.000112035	0.022417225	Promoter (<=1kb)	246782	ENSMUSG00000042709
H3K27ac	Npr1	8.62	7.67	0.379214165	0.000114491	0.022651276	Promoter (<=1kb)	18160	ENSMUSG00000027931
H3K27ac	Casp4	9.88	9.41	0.259939018	0.000120618	0.023421377	Promoter (<=1kb)	12363	ENSMUSG00000033538
H3K27ac	Slc22a15	9.66	9.99	-0.219420952	0.000122278	0.023421377	Promoter (<=1kb)	242126	ENSMUSG00000033147
H3K27ac	Sptssa	10.02	9.62	0.240796203	0.000122521	0.023421377	Promoter (<=1kb)	104725	ENSMUSG00000044408
H3K27ac	Reep4	9.57	9.09	0.26477621	0.000124596	0.023421377	Promoter (2-3kb)	72549	ENSMUSG00000033589
H3K27ac	Epc2	9.47	9.87	-0.23999768	0.000125785	0.023421377	Intron	227867	ENSMUSG00000069495
H3K27ac	Cast	10.33	9.95	0.232923415	0.00012737	0.023421377	Promoter (<=1kb)	12380	ENSMUSG00000021585
H3K27ac	Phactr2	8.81	9.30	-0.2639845	0.000128897	0.023421377	Intron	215789	ENSMUSG00000062866
H3K27ac	Oprd1	9.74	10.14	-0.238004121	0.000129025	0.023421377	Distal Intergenic	18386	ENSMUSG00000050511
H3K27ac	Jarid2	9.13	9.56	-0.246449331	0.000132579	0.023820887	Promoter (<=1kb)	16468	ENSMUSG00000038518
H3K27ac	Cep19	9.15	8.63	0.270348496	0.000140218	0.024885344	Promoter (<=1kb)	66994	ENSMUSG00000035790
H3K27ac	Akap8l	9.47	8.97	0.258737079	0.000141979	0.024885344	Promoter (<=1kb)	54194	ENSMUSG00000002625
H3K27ac	Ssbp2	9.14	9.62	-0.25947631	0.000142743	0.024885344	Exon	66970	ENSMUSG00000003992
H3K27ac	Mgat5	9.62	9.93	-0.212035169	0.000147986	0.02554643	Intron	107895	ENSMUSG00000036155
H3K27ac	Mtx1	9.63	8.65	0.36474642	0.000151176	0.0257936	Promoter (<=1kb)	17827	ENSMUSG00000064068
H3K27ac	Dram1	10.57	10.11	0.254647633	0.000152665	0.0257936	Promoter (<=1kb)	71712	ENSMUSG00000020057
H3K27ac	Lbr	10.10	9.59	0.261424275	0.000153812	0.0257936	Promoter (<=1kb)	98386	ENSMUSG00000004880
H3K27ac	Dnajb2	10.02	9.46	0.277478543	0.000155566	0.025841553	Promoter (<=1kb)	56812	ENSMUSG00000026203
H3K27ac	Itpr1	9.62	9.06	0.274614509	0.000157328	0.02588997	Promoter (<=1kb)	16438	ENSMUSG00000030102
H3K27ac	Ptprm	10.61	10.89	-0.195244633	0.000161511	0.026332293	Intron	19274	ENSMUSG00000033278

Table S2 cd.

Histone modification	SYMBOL	Conc_RSV	Conc_PBS	Fold	p.value	FDR	Genomic annotation	gene ID	ENSEMBL
H3K27ac	Zfp956	9.59	9.92	-0.212365344	0.000163948	0.026484336	Distal Intergenic	101197	ENSMUSG00000045466
H3K27ac	Dhx40	8.59	7.76	0.325955688	0.000166863	0.026710292	Distal Intergenic	67487	ENSMUSG00000018425
H3K27ac	Samhd1	9.77	9.22	0.266765225	0.000192378	0.030516987	Promoter (<=1kb)	56045	ENSMUSG00000027639
H3K27ac	Svil	11.80	11.98	-0.148540966	0.000196084	0.03082718	Promoter (<=1kb)	225115	ENSMUSG00000024236
H3K27ac	Fgd6	9.60	8.81	0.30992667	0.000201404	0.031196417	Promoter (<=1kb)	13998	ENSMUSG00000020021
H3K27ac	Magi2	8.05	8.65	-0.277080423	0.000202165	0.031196417	Intron	50791	ENSMUSG00000040003
H3K27ac	Spock1	8.53	9.01	-0.253288261	0.000203748	0.031196417	Distal Intergenic	20745	ENSMUSG00000056222
H3K27ac	Neu3	9.06	9.48	-0.231274364	0.000209046	0.031720034	3' UTR	50877	ENSMUSG00000035239
H3K27ac	Dlg5	8.67	9.11	-0.241070347	0.00021077	0.031720034	Promoter (<=1kb)	71228	ENSMUSG00000021782
H3K27ac	Id1	10.66	9.95	0.292960075	0.000213186	0.031811736	Promoter (<=1kb)	15901	ENSMUSG00000042745
H3K27ac	2700054A10Rik	9.00	9.50	-0.252142009	0.000220732	0.032501057	Promoter (<=1kb)	72578	ENSMUSG00000117042
H3K27ac	Vmn1r213	8.39	8.89	-0.254716776	0.00022232	0.032501057	Distal Intergenic	171249	ENSMUSG00000060024
H3K27ac	Lap3	9.83	8.84	0.330150405	0.000224494	0.032501057	Promoter (<=1kb)	66988	ENSMUSG00000039682
H3K27ac	Patl1	10.82	10.37	0.241313444	0.000225715	0.032501057	Promoter (<=1kb)	225929	ENSMUSG00000046139
H3K27ac	Usp42	9.08	7.98	0.340733909	0.00022805	0.032501057	Promoter (<=1kb)	76800	ENSMUSG00000051306
H3K27ac	Dixdc1	10.46	10.02	0.238163408	0.000232346	0.032501057	Promoter (<=1kb)	330938	ENSMUSG00000032064
H3K27ac	Gsk3b	12.00	12.20	-0.163044444	0.000232583	0.032501057	Promoter (<=1kb)	56637	ENSMUSG00000022812
H3K27ac	Gm13056	11.19	11.00	0.159923126	0.000233525	0.032501057	Promoter (<=1kb)	100503810	ENSMUSG00000085395
H3K27ac	Tanc2	9.03	9.47	-0.241238159	0.000234418	0.032501057	Intron	77097	ENSMUSG00000053580
H3K27ac	Prrc2c	10.43	10.73	-0.204614064	0.000239642	0.032946918	Promoter (<=1kb)	226562	ENSMUSG00000040225
H3K27ac	Kdm7a	8.97	9.45	-0.245033101	0.000241683	0.032946918	Exon	338523	ENSMUSG00000042599
H3K27ac	Dusp1	10.80	10.14	0.279660001	0.000243247	0.032946918	Promoter (<=1kb)	19252	ENSMUSG00000024190
H3K27ac	H2-T22	9.40	8.40	0.323957358	0.000251019	0.033740029	Promoter (<=1kb)	15039	ENSMUSG00000056116
H3K27ac	Inava	10.16	10.41	-0.182965844	0.000264167	0.03492163	Promoter (<=1kb)	67313	ENSMUSG00000041605
H3K27ac	Swap70	9.65	10.07	-0.232352585	0.000265456	0.03492163	Exon	20947	ENSMUSG00000031015
H3K27ac	Gm15506	9.34	8.66	0.281394598	0.00026576	0.03492163	Promoter (<=1kb)	100040769	NA
H3K27ac	Irf1	8.76	7.96	0.287285791	0.000279905	0.036507941	Promoter (<=1kb)	16362	ENSMUSG00000018899
H3K27ac	Ptpa	9.53	9.10	0.234623594	0.00028399	0.036768371	Promoter (<=1kb)	110854	ENSMUSG00000039515
H3K27ac	Kcnip4	8.40	8.86	-0.236212086	0.000289682	0.037231512	Intron	80334	ENSMUSG00000029088
H3K27ac	Nus1	9.92	9.38	0.25204511	0.000294669	0.037598026	Promoter (<=1kb)	52014	ENSMUSG00000023068
H3K27ac	Hnrnpa0	10.25	9.60	0.266946726	0.000304644	0.038591222	Promoter (<=1kb)	77134	ENSMUSG00000007836
H3K27ac	Gprc5a	10.16	9.78	0.21957316	0.000312908	0.039111515	Promoter (<=1kb)	232431	ENSMUSG00000046733
H3K27ac	Crebbp	9.32	9.78	-0.235492855	0.000313533	0.039111515	Exon	12914	ENSMUSG00000022521
H3K27ac	Fam149b	8.22	7.35	0.28743592	0.000317337	0.039111515	Promoter (<=1kb)	105428	ENSMUSG00000039599
H3K27ac	Pfn2	8.11	8.63	-0.247900932	0.000317637	0.039111515	Distal Intergenic	18645	ENSMUSG00000027805
H3K27ac	Evi5	9.15	9.52	-0.219474828	0.00032352	0.039559291	Promoter (<=1kb)	14020	ENSMUSG00000011831
H3K27ac	Zbtb7b	10.81	10.34	0.237466218	0.000326496	0.039647858	Promoter (<=1kb)	22724	ENSMUSG00000028042
H3K27ac	Irgm2	8.82	7.98	0.282203892	0.000339383	0.04093046	Promoter (<=1kb)	54396	ENSMUSG00000069874
H3K27ac	Gmpr	9.17	9.51	-0.209567553	0.000351437	0.041952647	3' UTR	66355	ENSMUSG00000000253
H3K27ac	Eif2ak3	10.38	9.71	0.262763887	0.000352623	0.041952647	Promoter (<=1kb)	13666	ENSMUSG00000031668
H3K27ac	Paip2b	9.73	9.08	0.25935961	0.000366454	0.043305569	Promoter (<=1kb)	232164	ENSMUSG00000045896
H3K27ac	Zfp708	9.68	9.01	0.2597219	0.000371279	0.04356128	Promoter (<=1kb)	432769	ENSMUSG00000058883
H3K27ac	Stard13	11.50	11.71	-0.159845264	0.000373566	0.04356128	Promoter (<=1kb)	243362	ENSMUSG00000016128
H3K27ac	Sned1	8.17	8.68	-0.240615379	0.000383519	0.044236221	Exon	208777	ENSMUSG00000047793
H3K27ac	Tapt1	11.93	12.15	-0.164727412	0.000384379	0.044236221	Promoter (<=1kb)	231225	ENSMUSG00000046985
H3K27ac	Abl1	9.45	9.93	-0.235068046	0.000401019	0.045712193	Exon	11350	ENSMUSG00000026842
H3K27ac	Txndc2	8.47	8.91	-0.224760024	0.000402396	0.045712193	Intron	213272	ENSMUSG00000050612
H3K27ac	Jdp2	10.03	9.72	0.200814476	0.00040612	0.045839532	Promoter (<=1kb)	81703	ENSMUSG00000034271
H3K27ac	Ubc	11.66	11.33	0.203890834	0.000411257	0.046123602	Promoter (<=1kb)	22190	ENSMUSG00000008348
H3K27ac	Vgll4	10.84	10.51	0.204714434	0.000415077	0.046257476	Promoter (<=1kb)	232334	ENSMUSG00000030315
H3K27ac	Zfp239	9.53	8.76	0.260634687	0.000423292	0.046876266	Distal Intergenic	22685	ENSMUSG00000042097
H3K27ac	Bmp3	9.45	9.79	-0.202544634	0.00042837	0.047142165	Distal Intergenic	110075	ENSMUSG00000029335
H3K27ac	Ift172	9.14	8.32	0.262642001	0.000441306	0.047826639	Promoter (<=1kb)	67661	ENSMUSG00000038564
H3K27ac	Foxp2	9.59	9.98	-0.214929525	0.000442487	0.047826639	Promoter (<=1kb)	114142	ENSMUSG00000029563
H3K27ac	Brpf3	9.07	7.72	0.268982481	0.000443473	0.047826639	Promoter (<=1kb)	268936	ENSMUSG00000063952
H3K27ac	Tmem253	10.62	10.14	0.230041043	0.000446866	0.047826639	Promoter (<=1kb)	619301	ENSMUSG00000072571
H3K27ac	Parp9	8.14	7.22	0.266491364	0.000448171	0.047826639	Distal Intergenic	80285	ENSMUSG00000022906
H3K27ac	Psmb9	8.51	7.47	0.270026385	0.000457337	0.048510805	Promoter (<=1kb)	16912	ENSMUSG00000096727
H3K27ac	Rpusd3	8.30	7.50	0.257607425	0.000468933	0.049442992	Promoter (<=1kb)	101122	ENSMUSG00000051169
H3K27ac	Firre	9.02	8.48	0.236036663	0.000475632	0.049850784	Exon	103012	ENSMUSG00000085396
